# A population code for spatial representation in the zebrafish telencephalon

**DOI:** 10.1038/s41586-024-07867-2

**Published:** 2024-08-28

**Authors:** Chuyu Yang, Lorenz Mammen, Byoungsoo Kim, Meng Li, Drew N. Robson, Jennifer M. Li

**Affiliations:** 1 Max Planck Institute for Biological Cybernetics, Tuebingen, Germany; 2 University of Tuebingen, Tuebingen, Germany; 3 Present Address: Department of Neurobiology and Behavior, Cornell University, Ithaca, NY USA; 4 Present Address: Shanghai Institute of Microsystem and Information Technology, Chinese Academy of Sciences, Shanghai, China; 5 Present Address: INSIDE Institute for Biological and Artificial Intelligence, Shanghai, China

**Keywords:** Neural circuits, Navigation

## Abstract

Spatial learning in teleost fish requires an intact telencephalon^1^, a brain region that contains putative analogues to components of the mammalian limbic system (for example, hippocampus)^2–4^. However, cells fundamental to spatial cognition in mammals—for example, place cells (PCs)^5,6^—have yet to be established in any fish species. In this study, using tracking microscopy to record brain-wide calcium activity in freely swimming larval zebrafish^7^, we compute the spatial information content^8^ of each neuron across the brain. Strikingly, in every recorded animal, cells with the highest spatial specificity were enriched in the zebrafish telencephalon. These PCs form a population code of space from which we can decode the animal’s spatial location across time. By continuous recording of population-level activity, we found that the activity manifold of PCs refines and untangles over time. Through systematic manipulation of allothetic and idiothetic cues, we demonstrate that zebrafish PCs integrate multiple sources of information and can flexibly remap to form distinct spatial maps. Using analysis of neighbourhood distance between PCs across environments, we found evidence for a weakly preconfigured network in the telencephalon. The discovery of zebrafish PCs represents a step forward in our understanding of spatial cognition across species and the functional role of the early vertebrate telencephalon.

## Abstract (web summary)

Using a tracking microscope for freely moving animals, the authors discover a population of place cells in the zebrafish brain and demonstrate that a non-amniote brain is capable of integrating allothetic and idiothetic information to create a neural map of space.

## Main

Animals generate an internal map of their environment through spatial exploration^6^, integrating multiple sources of information including landmarks, geometry and self-motion^9–15^. Over half a century of research in mammals has uncovered key computational building blocks of spatial cognition, beginning with the seminal discovery of place cells (PCs)^5^. Notably, PCs have been comprehensively identified only in the hippocampus of birds^16^ and mammals^5,17^. In teleost fish, which shared a common ancestor with mammals around 450 million years ago^18^, the location or even existence of the hippocampus is an area of active debate^2,19^, particularly given the lack of clear functional evidence for PCs^20–23^.

Several methodological challenges may have limited the discovery of PCs in fish. Electrophysiological recordings in freely moving adult fish have sampled only a small portion (under 1%) of cells in the telencephalon, a region hypothesized to contain the functional analogue of the mammalian hippocampus^1–3,19^. Although brain-wide imaging is possible in larval zebrafish^24,25^, most studies have used tethered preparations, which preclude naturalistic spatial exploration and can suppress spontaneous movement rate by roughly tenfold^7^. Mammalian studies have shown that PC activity is most robust during continuous running^26^. Thus, the suppressed movement rate of tethered larval zebrafish may have been an obstacle in the search for PCs.

To overcome these limitations we performed brain-wide imaging in freely swimming larval zebrafish using tracking microscopy^7,27^, and systematically characterized the spatial information content^8^ of each recorded neuron. In contrast to head direction cells and speed cells, which are both enriched in the fish rhombencephalon^7,28,29^, cells that encode spatial position (that is, PCs) are enriched in the fish telencephalon. Collectively these PCs can be used to decode the animal’s spatial location. By projection of this population code onto a two-dimensional activity manifold, we observe untangling of spatial representation across time, suggesting experience-dependent refinement of place codes.

Through an extensive series of environmental manipulations, we find that zebrafish PCs potentially integrate both allothetic and idiothetic information, flexibly remap to generate distinct spatial maps and exhibit some degree of preconfiguration^30,31^. Collectively these results suggest that the compact neural network of the zebrafish telencephalon can generate functional units similar to mammalian PCs, and offer a potential window into the evolution of spatial cognition across vertebrate species.

## Identification of cells encoding positional information

**Fig. 1:**
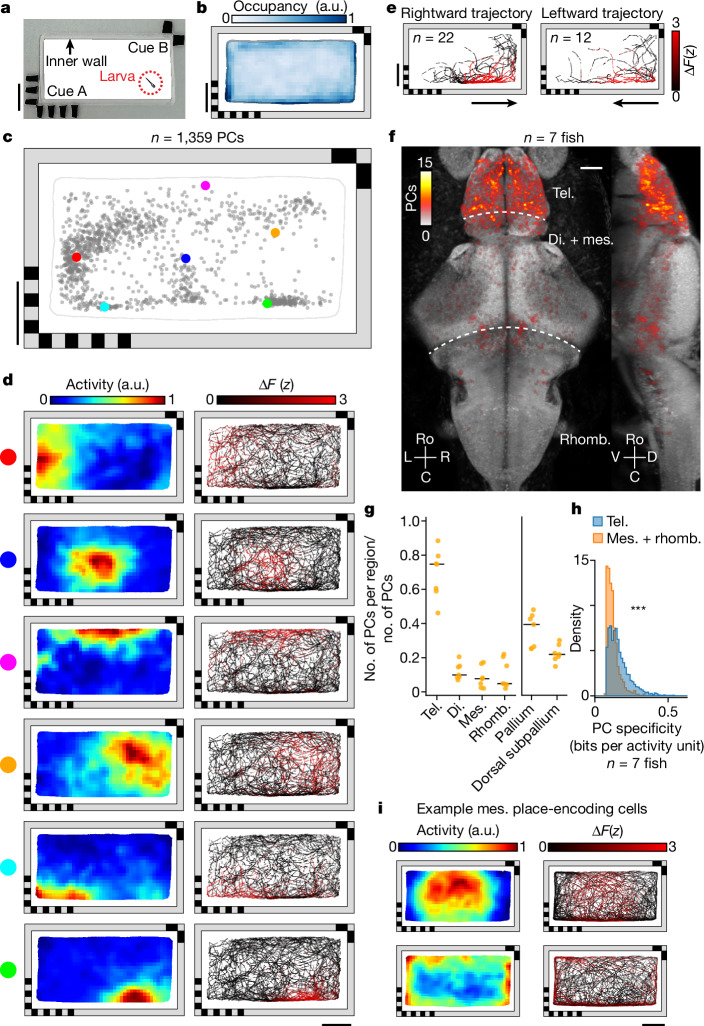
Identification of PCs in the larval zebrafish brain. **a**, Rectangular behavioural arena with two landmark cues containing different visual patterns on opposing corners. There is a transparent inner polydimethylsiloxane (PDMS) wall (1.5 mm wide) preventing the animal from directly contacting the landmarks. **b**, Normalized median chamber occupancy across seven animals. **c**, Distribution of PF COM for all PCs with a confined PF in an example fish (Methods). Each dot represents the COM of one cell’s PF (Methods). The six example cells (coloured dots) are shown in **d**. **d**, Spatial activity maps (left, averaged neural response by location; Methods) and corresponding animal trajectories (right, colour coded by neural activity) are shown for six example cells from **c**. **e**, Responses of an example cell with a non-directional PF for two orientations of traversal (leftwards and rightwards) through the PF. Traversals across the 90 min experiment are colour coded by neural activity. Orientation is classified by the animal’s heading as it enters the PF. **f**, Anatomical distribution of PCs across seven animals plotted on the reference brain (maximum projection; Methods). **g**, Fraction of all PCs identified anywhere in the brain that are found in each brain region. Each animal is shown individually (orange dots, *n* = 7 animals), along with the median across animals (black lines). Anatomical subdivisions of the telencephalon (pallium and dorsal subpallium; Extended Data Fig. 2b) are plotted separately. **h**, Comparison of spatial specificity for PCs found in the telencephalon and for those found in the mesencephalon and rhombencephalon (*n* = 7 animals, *P* = 8.4 × 10^−124^, one-sided Mann–Whitney *U*-test). **i**, Spatial activity maps and animal trajectories for two example mesencephalic cells with significant spatial information encoding the interior (top) and periphery of the chamber (bottom). Scale bars, 10 mm (**a**–**e**,**i**), 50 µm (**f**). a.u., arbitrary units. di., diencephalon; mes., mesencephalon; rhomb., rhombencephalon; tel., telencephalon. Source Data

We constructed a rectangular behavioural chamber (50 × 25 mm^2^) with landmark cues at two opposing corners (Fig. 1a and Methods). A transparent, 1.5-mm-wide inner wall prevents the animal from directly contacting the landmarks. Larval zebrafish expressing pan-neuronal H2B-GCaMP6s (6–8 days post fertilization) were placed in this chamber for 90 min of free exploration (Fig. 1b). During this time we recorded each animal’s brain-wide calcium activity at two volumes per second and then extracted fluorescence across time *F*(*t*) for each cell centroid in the brain using non-negative matrix factorization (NMF)^32^ (Methods). Based on baseline-corrected neural activity Δ*F*(*t*) and the position of the animal in space, we generated a two-dimensional spatial activity map for each cell, calculated its spatial information content normalized by its mean activity (that is, spatial specificity)^8^ and identified the centre of mass (COM) of its place field (PF) (Fig. 1c,d and Methods).

**Extended Data Fig. 1:**
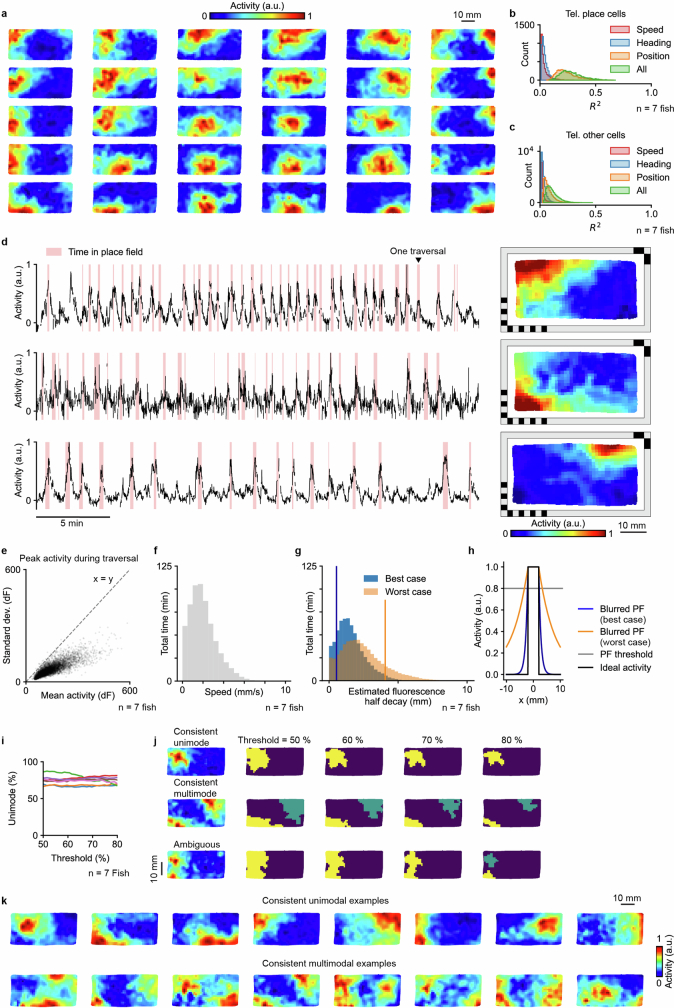
Place cell properties. **a**, Example spatial activity maps at different locations in the chamber. **b**, Modeling the relationship between activity and behavioral variables for telencephalic PCs. *R*² values of individual spatially tuned telencephalic cells after modeling their activity with ordinary least squares regression (OLS, **Methods**) using only the animal’s speed, heading, or two-dimensional position, as well as using all of them combined (n = 7 animals). **c**, Modeling the relationship between activity and behavioral variables for other cells in the telencephalon. **d**, Activity traces (left) and spatial activity maps (right) for three example PCs. The time that the fish spent inside the PF is marked in red. **e**, Statistics on the firing variability of PCs. One dot represents the mean and standard deviation of the peak activity across all traversals of one PC. A traversal is defined as a stay inside the PF that is interrupted only by departures shorter than 5 s. Data pooled across 7 animals. **f**, Distribution of swimming speed from the whole experiment from 7 animals, excluding quiescent time periods (swimming speed < 0.1 mm/s). **g**, Distribution of the estimated GCaMP6s fluorescence half decay distance in one linear dimension, based on the data from **f**, and assuming decay half-times of 0.56 s (10 Hz firing rate) and 1.21 s (80 Hz firing rate). Vertical lines indicate the mean - std. dev. for the best case and the mean + std. dev. for the worst case, based on the values of the distributions. **h**, Estimated actual PF blurring in one dimension based on an ideal PF with sharp edges (black line, −2.5 mm to 2.5 mm) and the best and worst cases of half decay distances shown in **g** (blue and orange lines). PF size is determined by the set of spatial bins with activity above 80% of the peak activity (with peak defined as 95^th^ percentile) of the spatial activity map. Blurring to each side is 0.17 mm (best case) and 1.31 mm (worst case). **i**, Percentage of unimodal PCs as a function of the threshold for defining the firing field. **j**, Spatial activity maps (left) and thresholded firing fields (right) at different thresholds for an example neuron with consistently unimodal firing field (top), a neuron with consistently multimodal firing field (middle), and an ambiguous neuron (bottom) with unimodal or multimodal firing field depending on the choice of threshold between 50% and 80%. **k**. More examples of neurons that are consistently unimodal (top) or consistently multimodal (bottom) as firing field threshold is swept between 50% and 80%.

For identification of cells with significant spatial specificity, we shuffled the activity of each cell 1,000 times by circular permutation (Methods). The shuffled data form a null distribution of spatial specificity for a given cell. A cell was designated as a PC if its spatial specificity was significantly greater than the null distribution generated from its shuffled activity (*z*-score ≥ 5) and the distribution of spatial specificity across all cells (*z*-score ≥ 3). With these significance criteria we identified 1,081 ± 329 (mean ± s.d.) PCs in each animal, with PFs distributed across the chamber (Fig. 1c,d, Extended Data Fig. 1a and Methods). The majority of these cells fire uniquely at a single location whereas some show multiple PFs (Extended Data Fig. 1i–k).

Using linear regression we independently confirmed that the activity of the identified PCs is primarily predicted by the animal’s position in space rather than by other behavioural parameters such as heading or speed (Extended Data Fig. 1b and Methods). Consistent with this, we find PC activity to be generally heading independent (Fig. 1e). Spatial position also accounts for a greater portion of activity variance in PCs compared with non-PCs in the telencephalon (*P* < 10^−5^, one-sided Mann–Whitney *U*-test; Extended Data Fig. 1c). The stability of spatial representation for a given cell can be further quantified by measurement of the spatial correlation between two halves of the experiment. PCs have an average spatial correlation of 0.59 ± 0.19 (median ± s.d.) across the 90 min experiment and are consistently activated on multiple traversals through the same spatial location (Extended Data Fig. 1d,e and Supplementary Video 1). We note that slow GCaMP sensor kinetics combined with animal motion are likely to blur and enlarge each PF by at least 0.17–1.31 mm (Extended Data Fig. 1f–h and Methods).

## PCs are enriched in the telencephalon of larval zebrafish

**Extended Data Fig. 2:**
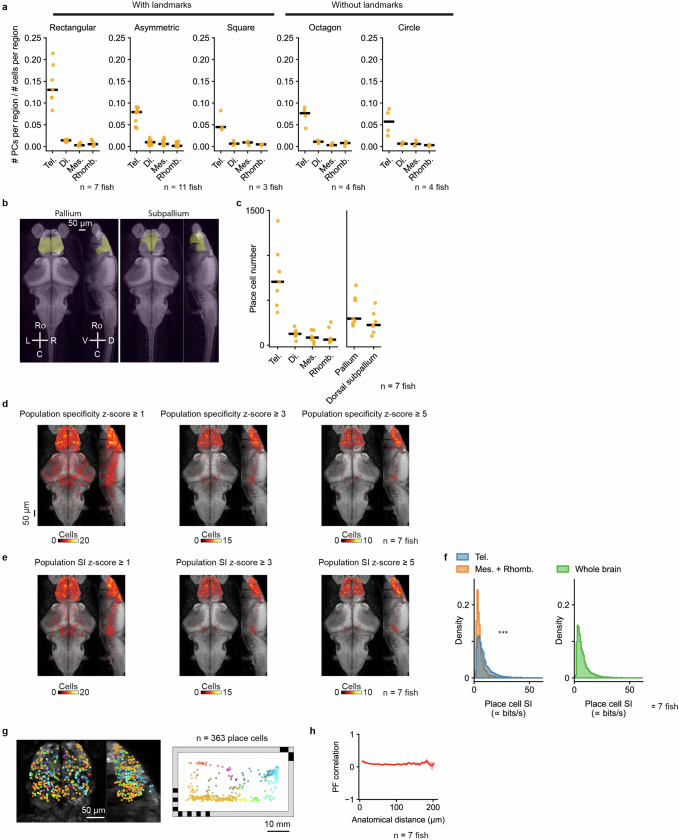
Anatomical analysis of place cells. **a**, Fraction of cells within each brain region that are classified as PCs, shown for five differently shaped behavioral chambers with or without landmarks: rectangle (Fig. 1a), asymmetric shape (Fig. 5a), square (Fig. 4e), octagon (Fig. 4i), and circle (Fig. 6a). **b**, Projections of telencephalic subdivisions: pallium and subpallium. Masks imported from the Z-Brain-Atlas. **c**, Number of PCs in each brain region. Each animal is shown individually (orange dots, n = 7 animals) along with the median across animals (black line). Tel., telencephalon; Mes., mesencephalon; Di., diencephalon; Rhomb., rhombencephalon. **d**, Anatomical distribution of cells that would be classified as PCs at different spatial specificity thresholds, based on a population specificity z-score ≥ 1 (left), ≥ 3 (middle), and ≥ 5 (right). Summation of 7 animals overlaid on a reference brain. The black bar indicates 50 μm. **e**, Anatomical distribution of cells that would be classified as PCs at different spatial information (SI) thresholds, based on a population SI z-score ≥ 1 (left), ≥ 3 (middle), and ≥ 5 (right). **f**, Comparison of SI for PCs found in the telencephalon and PCs found in the mesencephalon and rhombencephalon (left, n = 7 animals, *p* = 1.1 × 10^−98^, one-sided Mann–Whitney U test), as well as all PCs found in the whole brain (right, n = 7 animals). Since SI was calculated from calcium activity without attempting to convert to spikes/s, the SI values reported here are proportional to classic SI, measured in bits/s, but with an unspecified constant of proportionality. **g**, Anatomical distribution of PCs (left) colored by their PF locations (right). **h**, The relationship between anatomical distance and PF correlation. The solid line indicates the mean. The shaded region indicates the standard error of the mean. To avoid any potential caveat of imperfect cell merging along the dorsal-ventral axis, only cell pairs from the same horizontal plane are plotted. To avoid overestimating correlation due to shared measurement noise, adjacent cell pairs with NMF spatial footprints having any shared pixels are excluded (1.19% ± 0.54% of all cell pairs).

The anatomical distribution of spatial information in the brain is consistent across animals. We find that, in every animal, the telencephalon contains the highest number and fraction of PCs (69 ± 14%, mean ± s.d.; Fig. 1f,g and Extended Data Fig. 2a–c), regardless of chamber geometry or landmarks, whereas telencephalic neurons consist of only 8 ± 1% (mean ± s.d.) of the recorded neurons on average. This enrichment of PCs in the telencephalon can also be observed by simply ranking cells across the brain according to their spatial specificity (Extended Data Fig. 2d) or spatial information (Extended Data Fig. 2e). In every animal, cells with the highest-ranked spatial specificity are located primarily in the telencephalon (91 ± 9%, mean ± s.d., for the top 100 cells).

PCs that encode a given spatial location within the telencephalon are not clustered together anatomically (Extended Data Fig. 2g), with little correlation between each cell pair’s anatomical distance and the similarity of their spatial activity maps (Extended Data Fig. 2h).

Outside the telencephalon, cells with the highest spatial specificity are located in a small number of bilaterally symmetric neuronal clusters near the boundary between the mesencephalon and rhombencephalon (Fig. 1f). However, the spatial specificity or spatial information of these cells is significantly lower (*P* < 10^−5^, one-sided Mann–Whitney *U*-test) than for those within the telencephalon (Fig. 1h and Extended Data Fig. 2f). These non-telencephalic PCs appear to contain coarse-grained spatial information with larger PFs that often encompass the entire interior or border of the chamber (Fig. 1i), whereas the telencephalon contains finer-scale spatial information that forms a population code of space.

## The majority of spatially specific cells in the telencephalon are not consistent with the BVC model

**Extended Data Fig. 3:**
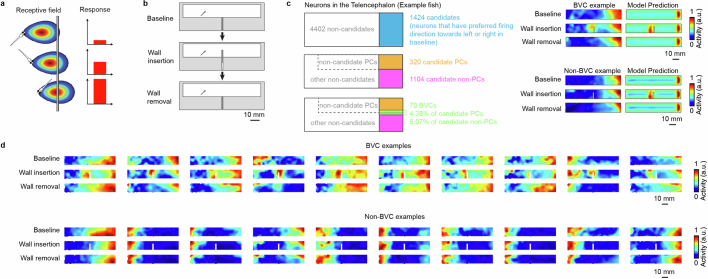
Boundary vector cell analysis. **a**, Schematic of the boundary vector cell (BVC) model, adapted from Lever et al.^33^. A BVC reaches the highest firing rate when the animal is at its preferred firing orientation and distance to the wall. **b**, Schematic of the experiment to test for BVCs in the larval zebrafish brain (**Methods**). A wall hidden in the middle of the rectangular chamber is inserted between the 1^st^ session (baseline) and the 2^nd^ session of the experiment. Then the wall is removed again for the recording of the 3^rd^ session of the experiment. The BVC model predicts that a BVC with a preferred horizontal firing orientation would duplicate its firing field when a new wall is inserted. **c**, Procedure for identification of boundary vector cells. First, we identified suitable candidate cells in the telencephalon with PFs parallel to the inserted wall (which could be PCs or non-PCs, left). We then fit the BVC model to the spatial activity maps of these candidate cells in session 1 and used the fitted model to generate predicted activity maps across sessions (with or without wall insertion). We compare the actual spatial activity maps (left) and the corresponding map predicted by the BVC model (right) to evaluate whether each cell is consistent with the BVC model. For each telencephalic candidate cell, the model is fit to the baseline (1^st^ session) and then used to predict the spatial activity map for the case of wall insertion or removal (2^nd^ and 3^rd^ sessions, **Methods**). Top right shows an example neuron that is consistent with the BVC model prediction. Bottom right shows an example of a neuron that does not follow the BVC model prediction. **d**, Additional examples of neurons that follow and do not follow the BVC model prediction.

A previous study has suggested that goldfish may use boundary vector cells (BVCs) rather than PCs for spatial navigation^20^, but no study of BVCs in zebrafish has yet been conducted. BVCs are defined by two unique functional signatures^33–35^: (1) each BVC fires at a preferred allocentric heading and distance to a boundary, and (2) when a border at a given BVC’s preferred heading is duplicated, its PF will also duplicate (Extended Data Fig. 3a). To test for BVCs, a movable wall was inserted and removed in the middle of an extended rectangular chamber across three sessions (Extended Data Fig. 3b and Methods). Given the direction of wall insertion, we identified a set of candidate telencephalic cells (1,641 ± 238 cells with PFs near the left or right wall of the chamber in session 1 (S1), mean ± s.d.), for which the BVC model predicts PF duplication in session 2 (S2) (Extended Data Fig. 3c and Methods). However, we find that only a small percentage of all candidate telencephalic cells (4.77 ± 0.14%, mean ± s.d.) and candidate telencephalic PCs (4.14 ± 0.31%, mean ± s.d.) fit the BVC model (Extended Data Fig. 3d), suggesting that, although BVCs exist in the zebrafish brain, they are distinct from PCs.

## Telencephalic PCs form a population code of space

**Fig. 2:**
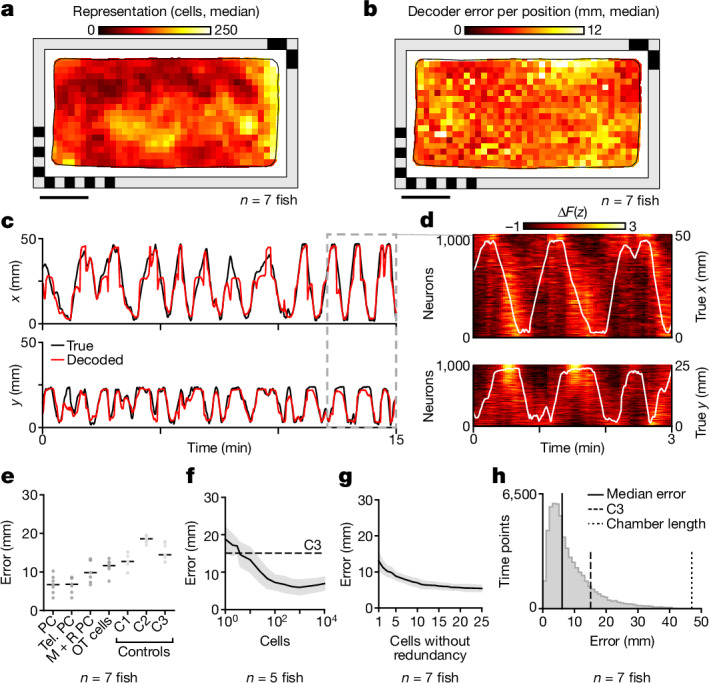
Physical location of larval zebrafish can be decoded from the population activity of PCs. **a**, Distribution of binarized telencephalic PFs in the chamber (median across animals). **b**, Median decoder error across animals at each bin within the chamber. To decode the physical location of the animal, a direct basis decoder was applied to telencephalic PCs (Methods). For all panels except **f**,**g**, the 1,000 cells with the highest spatial specificity were used as input to the decoder. **c**, Example traces show the *x* (top) and *y* (bottom) coordinates of the true (black) and decoded (red) animal locations. **d**, Activity of PCs across the final 3 min of **c**, together with true *x* (top) and *y* (bottom) coordinates of the animal (white overlay). Activity is shown two ways, sorting each cell by either the *x* or *y* component of its PF’s COM. **e**, Decoding performance for cells from different brain regions: PCs across the entire brain (PC), telencephalic PCs (Tel. PC), mes- and rhombencephalic PCs (M + R PC) and optic tectum cells (OT cells). Three controls are shown: random non-PCs (control 1, C1), uniformly random chamber positions (control 2, C2) and centroid of the animal’s spatial occupancy map (control 3, C3). Black horizontal bars represent median across animals. **f**, Decoder error as a function of the number of telencephalic cells included, in descending order of spatial tuning (Methods). Solid line indicates the mean and the shaded region indicates s.d. (the same applies to **g**). **g**, Decoder error as a function of the number of PCs included, using greedy selection to minimize redundancy (Methods). **h**, Distribution of decoder error across time (pooled across animals). Vertical solid line denotes median error, dashed line the behaviour-informed baseline and dotted line the accessible length of the chamber long axis. Scale bars, 10 mm. Source Data

**Extended Data Fig. 4:**
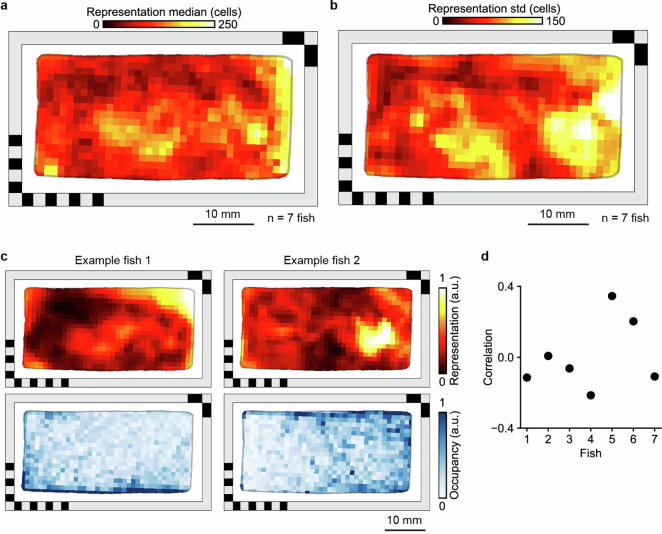
Spatial representation and occupancy. **a**, Distribution of telencephalic PFs encoding different locations in the behavioral arena (same as Fig. 2a, n = 7 animals, median across animals is shown). To quantify the density of PFs across space, each spatial activity map was binarized. **b**, same as **a**, but showing the standard deviation of spatial representation across the 7 animals. **c**, Spatial representation (top) and spatial occupancy (bottom) for two example fish. Representation and occupancy are both normalized. **d**, Correlation between spatial representation and occupancy for 7 different animals.

**Extended Data Fig. 5:**
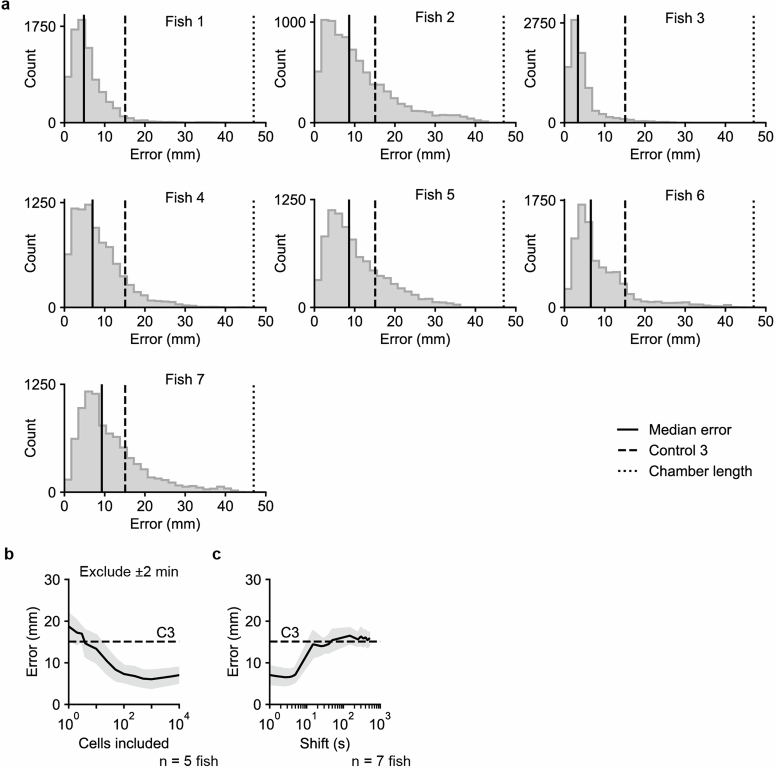
Additional quantification of decoder error. **a**, Distribution of decoder error for 7 animals. Vertical lines indicate the median error (black line), the behavior-informed baseline (15.08 mm, dashed line), and the accessible length of the chamber’s long axis (47 mm, dotted line). **b**, Replication of Fig. 2f but excluding from training the 2 min before and 2 min after the 1 min used for decoding (**Methods**). The solid line indicates the mean, and the shaded region indicates the standard deviation (the same applies to **c**). **c**, Decoder error as a function of timing shift between neural activity and animal position.

Collectively, telencephalic PCs span spatial locations throughout the behavioural chamber, with high variance in the degree of spatial representation of each location across animals (Fig. 2a and Extended Data Fig. 4a,b), and no apparent correlation between representational density and behavioural occupancy (Extended Data Fig. 4c,d). We applied a direct basis decoder^36^ to the population-level activity of PCs to determine whether we could predict the animal’s physical location in space (Methods). To avoid circularity, decoding was performed only on held-out time points not included in spatial activity map construction (Fig. 2, Extended Data Fig. 5 and Methods).

This linear decoder is able to predict the animal’s spatial location to within 6.69 ± 2.34 mm (median ± s.d.) using cells with the highest spatial specificity (up to the top 1,000) throughout the whole brain (Fig. 2b–e,h and Extended Data Fig. 5a). Using only telencephalic PCs, the decoder predicts spatial position with a similar degree of accuracy (6.82 ± 2.05 mm, median ± s.d.), which significantly outperforms decoding from PCs in both mesencephalon and rhombencephalon (9.84 ± 2.89 mm, median ± s.d.), cells in the optic tectum (11.63 ± 1.89 mm, median ± s.d.) and random cells across the brain (12.70 ± 2.30 mm, median ± s.d.) (Fig. 2e and Methods). The theoretical baseline decoding error is 15.08 ± 2.30 mm (median ± s.d.) and the optimal time lag for the decoder is 2.5 s (Extended Data Fig. 5c). The decoder reaches peak performance using around 1,000 cells with the highest spatial selectivity (Fig. 2f). However, because these cells may contain redundant encodings of position, we additionally demonstrate that, by selection of cells with non-redundant PFs, a decoding accuracy of 5.41 ± 0.81 mm (mean ± s.d.) can be achieved with a minimal set of 25 PCs (Fig. 2g and Methods).

## Telencephalic PCs form a spatial activity manifold that untangles over time

**Fig. 3:**
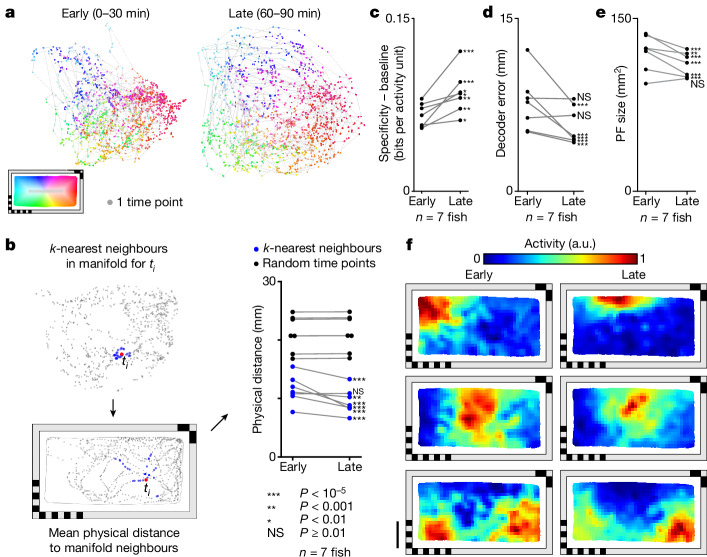
Activity manifold of PCs untangles across time. **a**, Change in manifold structure across two stages of the experiment (early, 0–30 min; late, 60–90 min). Individual time points are colour coded by the animal’s location within the behavioural arena. **b**, Mean physical distance to 30 neighbouring points in the two-dimensional manifold space, averaged across all time points (left). Change in this distance between early and late stages of the experiment (blue) and baseline (black, 30 randomly selected timepoints rather than neighbours; Methods). Each animal is shown individually. Changes within animals were tested using one-sided Mann–Whitney *U*-test. **c**, Change in mean spatial specificity across telencephalic PCs from early to late stages of the experiment. Mean spatial specificity of PCs is baseline corrected by subtracting the mean spatial specificity of an equal number of random cells. Each animal is shown individually. One-sided Mann–Whitney *U*-test is used to compare changes in PCs and those in random cells for individual animals. **d**, Change in mean decoder error from early to late stages of the experiment. Each animal is shown individually, and changes in decoder error within animals are tested using a one-sided Mann–Whitney *U*-test. **e**, Change in PF size from early to late stages of the experiment. Each animal is shown individually. Changes within animals are tested using one-sided Wilcoxon signed-rank test. **f**, Example spatial activity maps for early and late stages of the experiment. Each row is represents one PC. For the whole figure, telencephalic PCs used are defined across the experiment, excluding the first and last 15 min, and behavioural coverage was equalized between the two examined time intervals before making comparisons (Methods). Statistical significance ***P < 10^−5^, **P < 0.001, *P < 0.01, not significant (NS) *P* ≥ 0.01. See Supplementary Table 1 for exact *P* values of all statistical tests. Scale bar, 10 mm. Source Data

Across a 90 min session we found subtle changes in spatial representation during the early (first 30 min) and late (final 30 min) periods (Fig. 3). To control for potential differences in spatial coverage, we subsampled time points in both early and late periods to equalize their spatial coverage (Methods). When the population-level activity is embedded in a two-dimensional Isomap^37^, the spatial manifold becomes more untangled across time (Fig. 3a and Supplementary Video 2). To quantify this we define a metric, neighbourhood distance (Fig. 3b), that identifies for each time point *t*_*i*_ the 30 nearest time points in the embedded two-dimensional manifold space and measures their average distance to *t*_*i*_ in physical space. From the early to late period, the distance relationships in the activity manifold increasingly resemble those in the physical chamber, resulting in a significant decrease in neighbourhood distance (*P* < 10^−3^ for six of seven animals, one-sided Mann–Whitney *U*-test; Fig. 3b and Supplementary Table 1). Neighbourhood distance computed directly from neural activity without dimensionality reduction also shows a similar decrease across time (*P* < 10^−3^ for six of seven animals, one-sided Mann–Whitney *U*-test; Supplementary Table 1).

Untangling of the spatial activity manifold is accompanied by a significant increase in spatial specificity (*P* < 0.01 for seven of seven animals, one-sided Mann–Whitney *U*-test; Fig. 3c and Supplementary Table 1), a decrease in decoding error (*P* < 10^−5^ for five of seven animals, one-sided Mann–Whitney *U*-test; Fig. 3d and Supplementary Table 1) and a decrease in PF size (*P* < 10^−3^ for six of seven animals, one-sided Wilcoxon signed-rank test; Fig. 3e and Supplementary Table 1). Untangling of the activity manifold, along with the increased spatial specificity of individual cells (Fig. 3f), suggests refinement of the population code for spatial representation across time.

## Idiothetic information is a major input to spatial activity maps

**Fig. 4:**
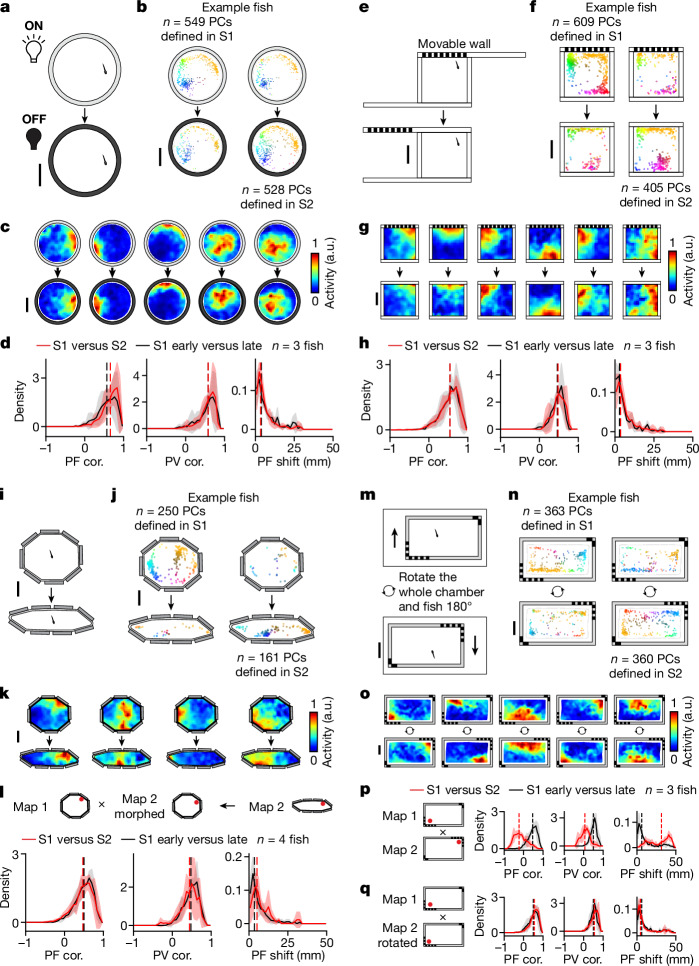
PC activity can be stable despite environmental changes when path integration is uninterrupted. **a**, Schematic of the light/dark experiment (Methods). **b**, Change in distribution of telencephalic PCs with confined PFs (Methods). PCs defined in S1 or S2 are plotted separately (left and right, respectively) for an example animal. Each dot represents the COM of one cell’s PF, colour coded by its position in the chamber (top, S1; bottom, S2). **c**, Example spatial activity maps (one cell per column). **d**, Spatial activity map correlation (PF cor.), population vector correlation (PV cor.) and shift in PF location (PF shift; Methods) are shown from left to right. Comparisons between S1 and S2 (red) are plotted together with control comparisons between the early (first half) and late (second half) periods of S1 (black). Solid lines denote mean distributions across all fish, shaded regions denote s.d. across all fish and vertical dashed lines represent medians of averaged distributions (the same applies to all histograms in this figure). **e**, Schematic of landmark-removal experiment (Methods). **f**–**h**, Analysis of landmark-removal experiment; **f**,**g**,**h** correspond to **b**,**c**,**d**, respectively. **i**, Schematic of wall-morphing experiment (Methods). **j**–**l**, Analysis of wall-morphing experiment; **j**,**k**,**l** correspond to **b**,**c**,**d**, respectively. To facilitate comparisons in **l**, we represent the activity of both sessions in terms of the spatial bins of S1 (see Methods for non-rigid morphing of the S2 spatial activity map). **m**, Schematic of chamber-rotation experiment. Between sessions the entire chamber including the fish is rotated 180° relative to the microscope (Methods). **n**,**o**, Analysis of chamber-rotation experiment; **n**,**o** correspond to **b**,**c**, respectively. **p**, Summary of map changes in telencephalic PCs across the two sessions by direct comparison of maps in the microscope reference frame. **q**, Similar to **l**, but the comparison is made in the chamber reference frame by rotating the spatial activity maps (Methods). Statistical tests for individual animals are summarized in Extended Data Fig. 6a. Scale bars, 10 mm. Source Data

**Extended Data Fig. 6:**
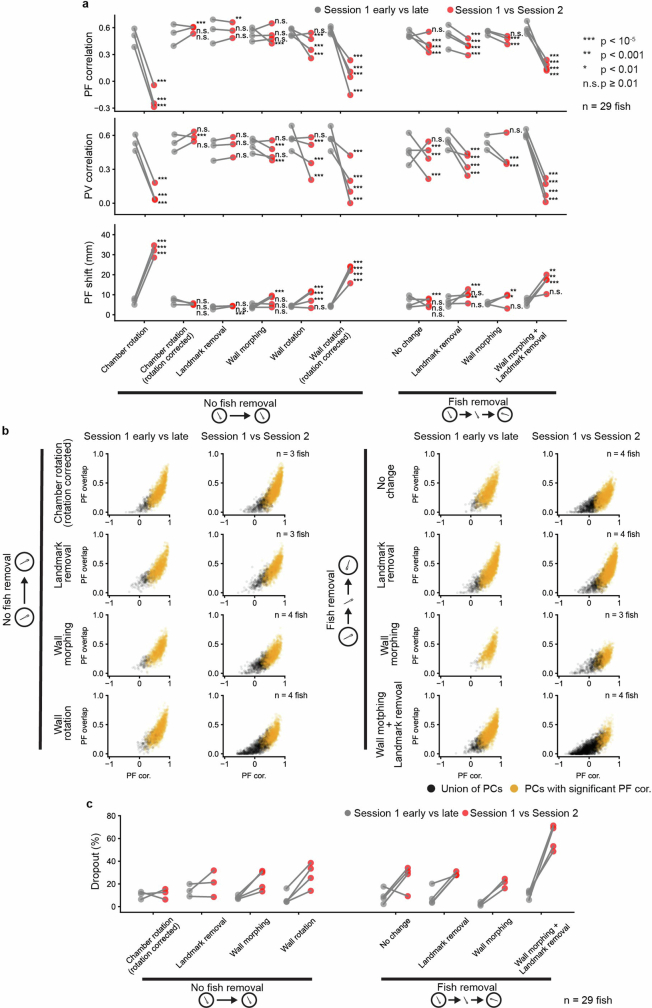
Summary of spatial activity map changes of telencephalic place cells under different environmental manipulations. **a**, The median of PF correlation (top), PV correlation (middle), and PF shift (bottom) for individual fish. Gray dots are comparisons between the early (1^st^ half) and late (2^nd^ half) stages of the 1^st^ session. The red dots are comparisons made between the 1^st^ session and the 2^nd^ session. Experiments from Figs. 4–6 are represented on the horizontal axis: chamber rotation (Fig. 4m), landmark removal (Fig. 4e), wall morphing (Fig. 4i), wall rotation (Fig. 5m), fish removal with no change in the chamber (Fig. 5a), landmark removal with fish removal (Fig. 5e), wall morphing with fish removal (Fig. 5i), and wall morphing with landmark removal and fish removal (Fig. 6a). Data from the same fish are connected by gray lines. The results of statistical tests are marked for each fish separately (one-sided Wilcoxon signed-rank test, *** for *p* < 10^−5^, ** for *p* < 0.001, * for *p* < 0.01, n.s. for *p* ≥ 0.01, see Supplementary Table 1 for exact *p*-values). **b**, The relationship between PF overlap and PF correlation for different experiments. Each dot represents a PC. PF overlap is defined as the intersection over union of its PFs in the two sessions or between the early and late half of session 1 (as control). Orange dots denote cells with significant PF correlation (one-sided shuffle test, *p* < 0.05). Briefly, to test the significance of the PF correlation, we circularly permuted (~ 1000 times, corresponding to the number of bins in the map) the observed spatial activity map of session 2 (or the 2^nd^ half of session 1 in controls), and recalculated the PF correlation across sessions for each permutation to construct the null distribution for a one-sided shuffle test. **c**, The dropout rates of PCs for individual fish (gray, within session control; red, cross-session comparison, as defined in **a**). A dropout cell is defined as a cell that does not retain significant PF correlation between sessions and is only classified as a PC in the first but not second session.

A hallmark of mammalian PCs is their ability to integrate information from both idiothetic and allocentric cues. Idiothetic information enables the animal to update its position in space based on path integration whereas an allocentric reference frame (that is, combination of landmarks and geometric boundaries) orients, constrains and stabilizes the animal’s internal position estimate^13,14^. To determine whether a non-visual source of information such as path integration significantly contributes to the spatial activity map, we adapted several classic mammalian paradigms to larval zebrafish (Fig. 4 and Extended Data Fig. 6): (1) transition from light to dark (Fig. 4a–d), (2) removal of landmarks (Fig. 4e–h) and (3) morphing of environmental boundaries (Fig. 4i–l). In all cases, if PCs are purely a read-out of visual patterns in the environments, then significant remapping should occur. Alternatively, because the animal was not removed from the chamber during these manipulations (that is, path integration was not interrupted), if PCs can rely on idiothetic information, then partial or no remapping is expected in all cases. Our results support the latter hypothesis (see below).

### Transition from light to dark

We recorded neural activity and behaviour for 60 min under bright diffuse white-light illumination (7.05 µW mm^−2^) in a circular chamber (S1), followed by 60 min with no white-light illumination in S2 (Fig. 4a and Methods). We note that a localized blue excitation spot (0.5 mm in radius) centred on the fish brain cannot be removed in these experiments due to its requirement for neural imaging (Methods). To quantify change in spatial representation we compute three metrics: (1) PF correlation, which is the correlation of spatial activity maps between sessions, (2) PF shift, which is the change in the COM of PFs across sessions and (3) population vector (PV) correlation, which is the correlation between population activity vectors at each spatial location (Methods). We found that PFs are retained despite substantial changes in visual illumination (Fig. 4b,c), as shown by a PF correlation of 0.66 (median, with interquartile range (IQR) 0.51–0.77; Methods), PV correlation of 0.58 (IQR 0.45–0.68) and PF shift of 3.38 mm (IQR 1.50–6.79 mm), all of which are not significantly different from control comparisons between the early and late periods of S1 (Fig. 4d; *P* values per fish using one-sided Wilcoxon signed-rank test are given in Supplementary Table 1).

### Landmark removal

**Extended Data Fig. 7:**
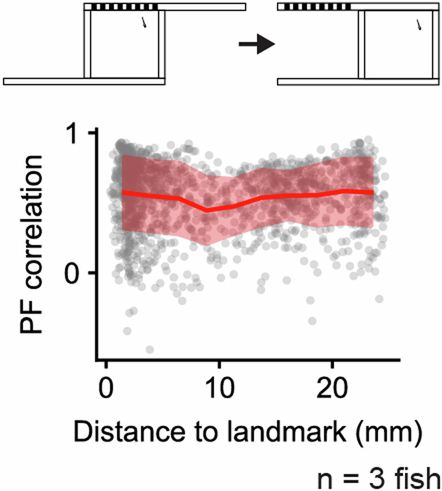
Place field correlation versus environmental features. Top, schematic of landmark removal experiment (Fig. 4e). Bottom, PF correlation between recording sessions in the landmark removal experiment as a function of the distance of the PF (represented by its COM) to the landmark. Red line indicates mean values after binning the data (n = 10 bins), red shaded region indicates standard deviation within bins.

To enable landmark removal without interruption of path integration we created a movable wall containing visual cues that can be remotely positioned (Fig. 4e). The wall was positioned with landmarks visible to the fish in S1 (75–90 min) and was then moved to hide the landmarks in S2 (60–100 min). We found that PFs are retained despite landmark removal (Fig. 4f,g), as shown by a PF correlation of 0.54 (IQR 0.36–0.67), PV correlation of 0.48 (IQR 0.37–0.58) and PF shift of 3.61 mm (IQR 1.45–7.48 mm), which are comparable to within-session controls (see Fig. 4h, Extended Data Fig. 6a and Supplementary Table 1 for *P* values). We did not observe a greater change in PF correlation for cells with PFs near landmarks (Extended Data Fig. 7).

### Morphing of environmental boundaries

**Extended Data Fig. 8:**
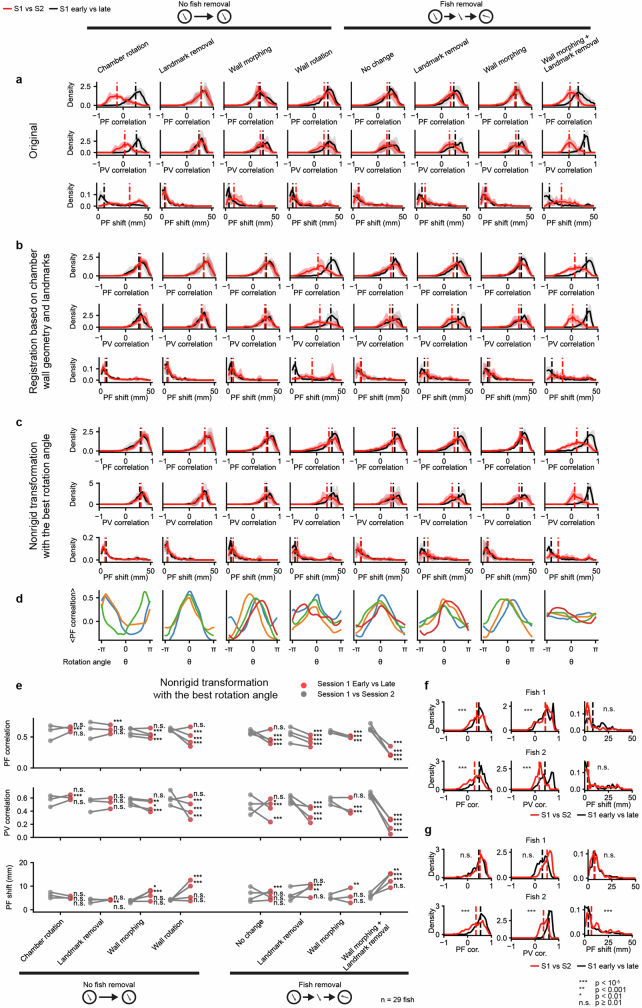
Additional quantification of map comparisons. **a**, Summary of the changes in telencephalic PCs by directly comparing the maps across two sessions in the microscope reference frame. Experiments from Fig. 4 and Fig. 5 are represented on the horizontal axis: chamber rotation (Fig. 4m), landmark removal (Fig. 4e), wall morphing (Fig. 4i), wall rotation (Fig. 5m), fish removal with no change in the chamber (Fig. 5a), landmark removal with fish removal (Fig. 5e), wall morphing with fish removal (Fig. 5i), and wall morphing with landmark removal and fish removal (Fig. 6a). From top to bottom are place field correlation (PF correlation), population vector correlation (PV correlation), and the shift in the place field location (PF shift, **Methods**). The union of telencephalic PCs from both sessions was used. The comparison between the 1^st^ and the 2^nd^ sessions (red) is plotted against a reference comparison between the early (1^st^ half) and late (2^nd^ half) stages of the 1^st^ session (black). Solid lines are the mean distributions across all fish, and the shaded region indicates the standard deviation across all fish. Vertical dashed lines are the medians of the averaged distributions. **b**, similar to **a** but after registering the maps from the 2^nd^ session to the 1^st^ session with reference to the chamber wall geometry and landmarks (**Methods**). **c**, similar to **b** but the transformation is done by finding the rotation of the reference anchor points that maximizes the mean PF correlation after registration (**Methods**). **d**, The mean PF correlation as a function of the anchor rotation angle used in the nonrigid transformation, for different experiments (**Methods**). **e**, Summary of changes in the spatial activity maps of telencephalic PCs under different environmental manipulations after applying the nonrigid transformation with the best rotation angle. The median of PF correlation, PV correlation, and PF shift are plotted for each fish. Red dots are comparisons made between the 1^st^ session and the 2^nd^ session. Gray dots are reference comparisons made between the early (1^st^ half) and late (2^nd^ half) stages of the 1^st^ session (also after nonrigid transformation with the best rotation angle). The results of statistical tests are marked for each fish separately (one-sided Wilcoxon signed-rank test, *** for *p* < 10^−5^, ** for *p* < 0.001, * for *p* < 0.01, n.s. for *p* ≥ 0.01). See Supplementary Table 1 for exact *p*-values. **f**, Analysis of fish removal experiment as in Fig. 5d, but only for individual fish where the olfactory cues were cleaned thoroughly before each recording session with soap and isopropanol. The results of statistical tests for different measures against a control comparison between the early (1^st^ half) and late (2^nd^ half) period of the 1^st^ session are shown for each fish (one-sided Wilcoxon signed-rank test, same for Extended Data Fig. 8g, fish 1 and fish 2 corresponds to fish 1 and fish 2 in Supplementary Table 1). **g**, Analysis of fish removal experiment as in Fig. 5d, but only for individual fish where the bottom was rotated without cleaning to scramble any potential olfactory cues (fish 1 and fish 2 correspond to fish 3 and fish 4 in Supplementary Table 1). See Supplementary Table 1 for exact *p*-values.

Next we asked whether the retention of spatial tuning properties can be explained by the interaction of idiothetic cues and boundary conditions, as previously observed in mammals^38^. To test this we created a morph chamber with eight flexibly connected segments that can be remotely adjusted without removing the animal (Methods and Fig. 4i). We found that PFs mostly maintain their position relative to each other across sessions (Fig. 4j,k) but not their absolute position in the chamber (Extended Data Fig. 8a and Supplementary Table 1). The absolute change in PF position across the two sessions is 8.41 mm (IQR 4.29–12.98 mm) whereas the relative change in PF position (obtained following application of a non-rigid transformation of S2 spatial activity maps to match the shape of the S1 chamber; Methods) is 5.13 mm (IQR 2.75–10.11 mm; Fig. 4l and Extended Data Figs. 6a and 8b). PF and PV correlations across sessions are similar to within-session controls (Fig. 4l, Extended Data Fig. 6a and Supplementary Table 1).

### Whole-chamber rotation as a control for extrinsic cues

To exclude the possibility that the retention of spatial maps in the above experiments is solely due to extrinsic cues outside of the behaviour chamber (for example from the imaging room or microscope), we performed the following control. After an initial imaging session (S1, 90 min), we rotated the entire chamber as a rigid body by 180° (S2, 60 min; Fig. 4m and Methods). Crucially, during this manipulation both the fish and chamber rotate together as one rigid body (that is, allothetic cues within the chamber and idiothetic information remain aligned).

If extrinsic cues are the dominant input to PCs, one would expect spatial activity maps to rotate by 0°; if not, they should rotate by 180°. Our results strongly support the latter hypothesis (Fig. 4n–q). Direct comparison of spatial activity maps (assuming 0° rotation) across the two sessions results in a PF correlation of −0.23 (IQR −0.44 to 0.03), PV correlation of 0.06 (IQR −0.1 to 0.2) and PF shift of 31.47 mm (IQR 15.59–39.49 mm) (Fig. 4p, Extended Data Fig. 6a and Supplementary Table 1). By contrast, following the application of 180° rotation to the spatial activity map in S2, PF correlation increased to 0.55 (IQR 0.39–0.68), PV correlation increased to 0.55 (IQR 0.41–0.66) and PF shift decreased to 4.52 mm (IQR 1.87–12.10 mm), all of which became indistinguishable from within-session controls (Fig. 4q, Extended Data Figs. 6a and 8b, and Supplementary Table 1).

## Allocentric visual information enables recovery of spatial activity maps

**Fig. 5:**
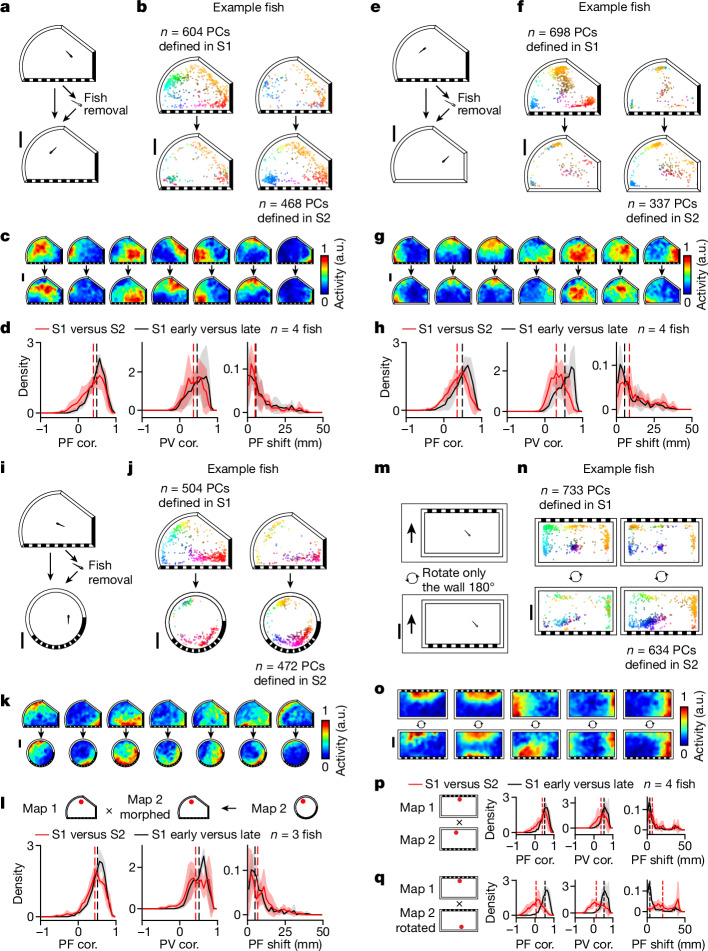
Allothetic visual information contributes to PC activity. **a**, Schematic of the fish-removal experiment. The fish was removed from the chamber to break path integration between recording sessions performed in the same asymmetric chamber with landmarks (Methods). **b**–**d**, Analysis of fish-removal experiments as in Fig. 4b–d, with **b**,**c**,**d** representing the same analyses in both figures. For all histograms, solid lines indicate mean distributions across all fish, shaded regions indicate s.d. across all fish and vertical dashed lines indicate medians of averaged distributions. **e**, Schematic of the landmark-removal experiment with fish removal. Following the first recording session in an asymmetric chamber with landmarks, the fish was removed and transferred to a chamber with the same geometry but no landmarks for the second recording session (Methods). **f**–**h**, Analysis of landmark-removal experiment with fish removal; **f**,**g**,**h** correspond to Fig. 4b–d, respectively. **i**. Schematic of the wall-morphing experiment with fish removal. Following the first recording session in an asymmetric chamber with landmarks, the fish was removed and transferred to a chamber with the same landmarks but a different geometry for the second recording session (Methods). One out of three fish went in the reverse direction—it was transferred from the circular chamber to the asymmetric chamber. **j**–**l**, Analysis of wall-morphing experiment with fish removal as in Fig. 4j–l, respectively. **m**, Schematic of wall-rotation experiment without fish removal. In comparison to the experiment shown in Fig. 4m, here only the chamber wall, but not the fish, is rotated 180° relative to both the fish and microscope (Methods). **n**–**q**, Analysis of wall-rotation experiment as in Fig. 4n–q, respectively. The results of statistical tests for individual animals are summarized in Extended Data Fig. 6a. Scale bars, 10 mm. Source Data

Next we asked whether, following interruption of path integration (for example, by removal of the animal from the chamber), a larval zebrafish can recover its previous spatial map based on allothetic cues^39,40^. We performed a series of experiments with either complete or partial retention of visual and geometric features across sessions (Fig. 5 and Extended Data Fig. 6): (1) no change in landmarks or geometry (Fig. 5a–d), (2) landmark removal without changing geometric features (Fig. 5e–h) and (3) morphing of geometric boundaries without changing landmarks (Fig. 5i–l). We recorded for 60–90 min in S1, removed the animal from the chamber for several minutes and then placed it in either the same chamber following cleaning (Methods) or a new chamber with partially altered landmarks or geometry for S2 (45–100 min). To interrupt path integration, animals were returned to the chamber at a different location and heading from where they were removed. We found nearly complete or partial recovery of spatial maps in all cases.

### No change in landmarks or geometric features

When animals were returned to the same chamber following removal or scrambling of any potential olfactory cues (Fig. 5a, Extended Data Fig. 8f,g and Methods), we found that the spatial map is mostly maintained between S1 and S2 despite the interruption in path integration (Fig. 5b–d and Extended Data Fig. 6a), as shown by a PF correlation of 0.40 (IQR 0.17–0.57), PV correlation of 0.36 (IQR 0.19–0.51) and PF shift of 5.05 mm (IQR 2.13–11.76 mm). PF shift was not significantly different from within-session control in most animals, although PF and PV correlation showed small but significant decreases (Fig. 5d, Extended Data Fig. 6a and Supplementary Table 1).

### Landmark removal or boundary morphing

Following landmark removal without changing chamber geometry, we found a PF correlation of 0.35 (IQR 0.16–0.50), PV correlation of 0.30 (IQR 0.16–0.44) and PF shift of 8.58 mm (IQR 4.17–17.79 mm; Fig. 5h), all of which show small but significant changes compared with within-session controls in most animals (Extended Data Fig. 6a and Supplementary Table 1). We found similar partial recovery of the spatial map following geometric morphing without changing landmarks. In this case we performed non-rigid transformation of the spatial activity maps in S2 to align the maps across sessions (Fig. 5l and Methods), resulting in a PF correlation of 0.44 (IQR 0.27–0.59), PV correlation of 0.42 (IQR 0.24–0.62) and PF shift of 6.77 mm (IQR 2.21–11.69 mm) (Fig. 5l, Extended Data Figs. 6a and 8b, and Supplementary Table 1).

## Conflict between sensory inputs

If both self-motion and visual information are potential inputs to PCs, we can ask which dominates when the two information streams come into conflict. To do this we created a chamber wall that can be remotely rotated without removal of fish or disassembly of the water-sealed chamber (Fig. 5m and Methods). Unlike whole-chamber rotation (Fig. 4m), the fish itself is not rotated across sessions (45–90 min for S1 and 45–75 min for S2), but only the walls of the chamber, along with the visual landmarks on the walls, undergo 180° rotation.

If idiothetic information predominates over allothetic information, the spatial map in S2 should directly correspond to that in S1; if the reverse is true, the spatial map in S2 should rotate by 180°. The result is intermediate between these two extremes (Fig. 5n–q). Direct comparison of spatial maps across sessions results in a PF correlation of 0.38 (IQR 0.15–0.53), PV correlation of 0.37 (IQR 0.18–0.53) and PF shift of 6.85 mm (IQR 3.12–17.48 mm), all of which show small but significant changes compared with within-session controls in most animals (Fig. 5p, Extended Data Fig. 6a and Supplementary Table 1). However, the application of 180° rotation to the spatial map in S2 results in an even lower PF correlation of 0.05 (IQR −0.2 to 0.25) and PV correlation of 0.12 (IQR −0.1 to 0.39), and an even higher PF shift of 20.47 mm (IQR 12.31–36.23 mm) (Fig. 5q, Extended Data Figs. 6a and 8b, and Supplementary Table 1). Thus the spatial map in S2 shows greater direct (rather than 180° rotated) correspondence to that in S1, suggesting that idiothetic information potentially predominates over allothetic visual information when the two information sources come into conflict.

## Representational flexibility of the PC network

### PCs demonstrate the potential for significant remapping across distinct environments

**Fig. 6:**
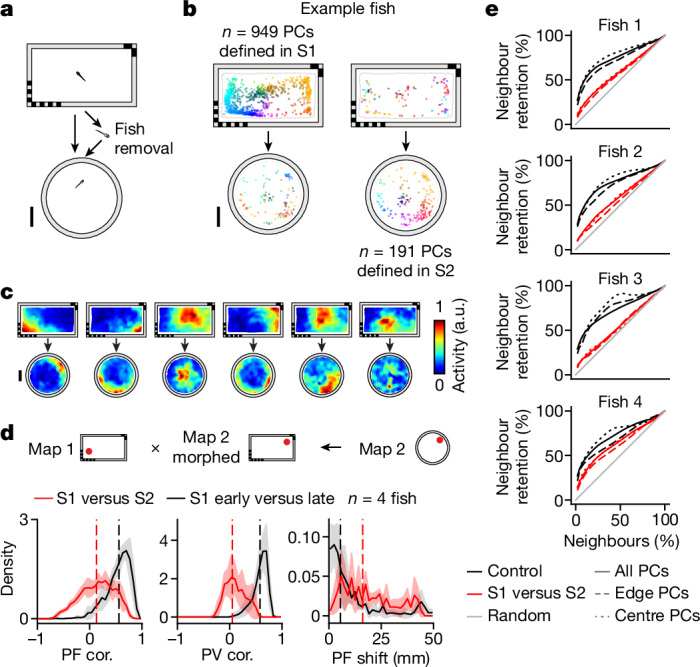
PCs exhibit representational flexibility across distinct environments. **a**, Schematic of the experiment combining landmark removal, wall morphing and fish removal. Following S1, the fish was removed and transferred to a chamber with a different geometry and no landmarks for S2 (Methods). **b**–**d**, Analysis of experiment performed as in Fig. 4j–l, respectively. **d**, For all histograms, solid lines indicate mean distributions across all fish, shaded regions indicate s.d. across all fish and vertical dashed lines indicate medians of averaged distributions. Results of statistical tests for individual animals are summarized in Extended Data Fig. 6a. **e**, We quantify the degree to which neighbourhood relationships between telencephalic PCs are maintained across sessions (‘neighbour retention %’; Methods). For each PC, neighbours are defined as cells with the highest PF correlation to the cell of interest, with a systematically varied inclusion threshold from the top 2% to the top 100%. Neighbour retention % is defined as the number of PCs that remain neighbours in S2 divided by the number of original neighbours in S1. The average neighbour retention % across PCs between S1 and S2 (solid red line) is plotted against the control comparison between the early and late stages of S1 (solid black line), as well as against comparisons with shuffled data (solid grey line). We also subdivided neurons into two groups based on the distance of each place field to the edge of the chamber in S1 (dashed lines represent distance to edge 3 mm or less, 233 ± 135 cells, mean ± s.d.; dotted lines represent distance to edge greater than 3 mm, 352 ± 171 cells, mean ± s.d.). Scale bars, 10 mm. Source Data

Mammalian PCs are notable for their representational flexibility^10,40,41^, while other spatial cells exhibit more coherent shifts across environments^42–44^. To test this in zebrafish we recorded the animal in S1 (90 min), removed it from the chamber to interrupt path integration and placed it in a new chamber with completely distinct geometry and visual features for S2 (60–80 min) (Fig. 6a–c). Following application of a non-rigid transformation to the spatial activity maps in S2 for alignment of these across sessions (Methods), we found a PF correlation of 0.13 (IQR −0.13 to 0.36), PV correlation of 0.05 (IQR −0.07 to 0.21) and PF shift of 16.13 mm (IQR 7.96–26.83 mm), all of which are significantly different from within-session controls, which had a PF correlation of 0.56 (IQR 0.39–0.68), PV correlation of 0.58 (IQR 0.48–0.65) and PF shift of 5.49 mm (IQR 2.03–12.46 mm) (Fig. 6d, Extended Data Figs. 6a and 8b, and Supplementary Table 1), demonstrating significant spatial remapping across distinct environments.

### Recovery of a previously stored map following remapping

**Extended Data Fig. 9:**
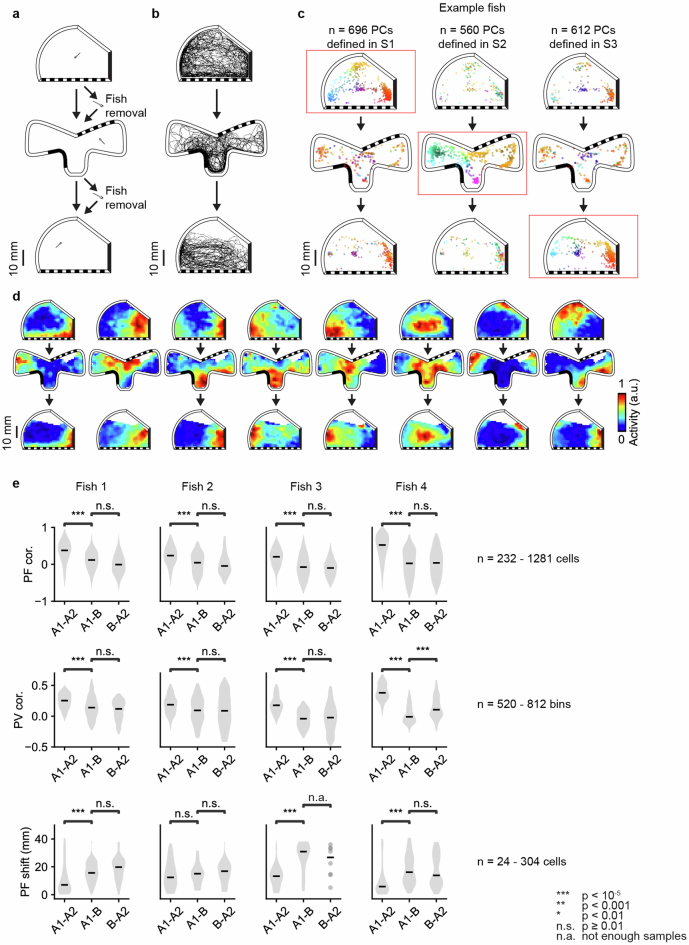
ABA experiment. **a**, Schematic of ABA experiment. After the 1^st^ recording session in an asymmetric chamber with landmarks, the fish was removed and transferred into a chamber with a distinct arrangement of geometric features and landmarks for the 2^nd^ recording session. For the 3^rd^ recording session, the fish was removed and transferred back to the chamber that was used in the 1^st^ recording session (**Methods**). **b**, Example animal trajectories in each session. **c**, Change in the distribution of telencephalic PCs with confined PFs, similar to Fig. 4b. Here, the distributions are plotted separately for 1^st^ session (top), 2^nd^ session (middle), and 3^rd^ session (bottom) for an example animal. Red boxes indicate the session where telencephalic PCs were defined in each column. **d**, Example spatial activity maps as Fig. 4c. **e**, Summary of the changes in telencephalic PCs across the 1^st^ and 2^nd^ session, the 2^nd^ and 3^rd^ session, and the 1^st^ and 3^rd^ session. Similar to Fig. 4d, PF cor., PV cor., and PF shift were calculated for 4 fish. Each distribution uses the union of telencephalic PCs from the two sessions being compared. Grey shaded regions show the distribution of each measurement, and black horizontal bars indicate medians. Each column is one fish. The results of statistical tests are marked on top of the distributions being compared (one-sided Mann–Whitney U test, *** for *p* < 10^−5^, ** for *p* < 0.001, * for *p* < 0.01, n.s. for *p* ≥ 0.01, n.a. for not enough samples). The range of sample sizes are provided on the right of each metric. See Supplementary Table 1 for exact *p*-values and exact sample sizes.

Once remapping occurred and a new spatial map was generated, we asked whether larval zebrafish can recover the spatial map of a previously encountered environment. In a three-session experiment, the animal was placed in chamber A (40–80 min), removed and placed in chamber B (30–60 min) and then returned to chamber A (40–60 min; Extended Data Fig. 9a,b). We refer to this as an ABA experiment. Chambers A and B have a distinct arrangement of geometric features and landmarks. A non-rigid transformation was performed on the spatial activity map in S2 to align maps across sessions (Methods). Following application of non-rigid transformation we found significant remapping between A and B, similar to the experiment in Fig. 6a, as shown by a PF correlation of 0 (IQR −0.26 to 0.24), PV correlation of 0.01 (IQR −0.12 to 0.15) and PF shift of 15.94 mm (IQR 10.08–26.96 mm) (Extended Data Fig. 9c–e and Supplementary Table 1). After returning to chamber A, fish showed partial recovery of spatial maps as shown by a PF correlation of 0.28 (IQR 0.06–0.49), PV correlation of 0.22 (IQR 0.10–0.34) and PF shift of 9.27 mm (IQR 3.31–16.84 mm; Extended Data Fig. 9c–e and Supplementary Table 1).

### Evidence for latent ensemble structure across environments

An active area of debate is the extent to which pairwise relationships between PCs are retained during remapping and whether animals can use similar maps to generalize across distinct environments^31,40^. When both environmental geometry and landmarks are altered, and path integration is interrupted, we find PF and PV correlation near 0 across sessions (Fig. 6d). Using these remapping experiments, we then asked whether there are latent local or global spatial relationships that are maintained across sessions despite substantial remapping. First we assessed whether local neighbourhood relationships between PCs are retained by computing the percentage of neurons that remain neighbours across sessions (Fig. 6e and Methods). To avoid the possibility of confounds due to overlapping fluorescent signal between adjacent cells, we restricted the analysis to pairs of cells with a minimum anatomical distance of over 20 µm. In all animals the neighbour retention percentages are significantly lower than controls (early versus late period of the same session, *P* < 10^−5^, four of four fish, one-sided Wilcoxon signed-rank test; Supplementary Table 1), suggesting a significant disruption of neighbourhood relationships during remapping. However, the percentage of neurons that remain neighbours is also significantly higher than by random chance (Fig. 6e; *P* < 10^−5^, four of four fish, mean percentage of remaining neighbours compared with 1,000 random shuffles; Methods), suggesting that local relationships between neurons are partially maintained.

**Extended Data Fig. 10:**
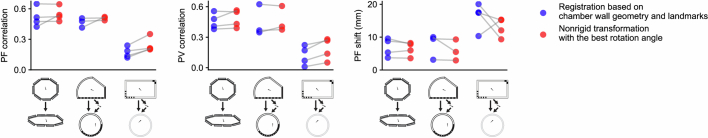
Weakly coherent place field rotations during remapping. The increase in the median PF correlation (left), PV correlation (middle), and PF shift (right) are shown for 3 experiments: wall morphing without fish removal (Fig. 4i), wall morphing with fish removal (Fig. 5i), and wall morphing with landmark removal and fish removal (Fig. 6a). Blue dots show the comparison of session 1 and session 2 after registration of session 2 maps based on session 1 wall geometry and landmarks. Red dots show the comparison of session 1 and session 2 after registering session 2 maps to session 1 maps using the nonrigid transformation with the best rotation angle. Data from the same fish are connected by gray lines. The observed improvement in PF correlation from map rotation was evaluated by a non-parametric shuffle test (**Methods**), applied to each fish individually: wall morphing without fish removal (1/4 fish, *p* < 10^−5^, 3/4 fish, *p* ≥ 0.01), wall morphing with fish removal (1/3 fish, *p* < 10^−5^, 2/3 fish, *p* ≥ 0.01), and wall morphing with landmark removal and fish removal (2/4 fish, *p* < 10^−5^, 1/4 fish, *p* < 0.01, 1/4 fish, *p* ≥ 0.01). See Supplementary Table 1 for exact *p*-values.

Second, we asked whether there are latent global relationships between spatial activity maps (for example, coherent PF rotations^31^). We synthetically applied 72 potential rotations (in 5° increments; Methods) to the spatial activity map in S2 and then used non-rigid transformation to align spatial activity maps across sessions (Extended Data Fig. 8d). We identified the rotation that maximized the correlation between the two spatial activity maps and found this to be variable across the four animals (131, −147, −86 and −100°). Application of optimal rotation and non-rigid transformation of S2 spatial activity maps slightly improved median PF correlation, PV correlation and PF shift, by 0.072 ± 0.036, 0.066 ± 0.032 and 3.35 ± 6.20 mm (all mean ± s.d.), respectively. Although the small improvement in PF correlation is higher than that expected by random chance (Extended Data Fig. 10, Methods and Supplementary Table 1), PF and PV correlation and PF shift all remain significantly different than within-session controls (Extended Data Fig. 8c,e and Supplementary Table 1), suggesting that global coherence between PCs is at most weakly maintained during remapping.

## Discussion

The previous lack of evidence for PCs outside of mammalian and avian species suggests either that the neural architecture needed to generate PCs evolved following the emergence of land vertebrates^5,16^ or that PCs in species such as teleost fish have not yet been discovered, potentially due to methodological limitations (for example, sparse recordings or tethered preparations). To overcome these challenges we recorded neural activity across the brain of freely swimming larval zebrafish using tracking microscopy^7^, and identified a population of PCs that are enriched in the zebrafish telencephalon and collectively encode the animal’s position in space.

Zebrafish PCs exhibit two functional hallmarks of mammalian PCs: (1) multimodal integration of information and (2) flexible representation of the environment. First, to demonstrate that zebrafish PCs potentially integrate multiple sources of sensory information^13,14^, we adapted some of the classic mammalian paradigms for manipulation of idiothetic or allothetic information to larval zebrafish (for example, transition from light to dark, manipulation of environmental features with or without interruption of path integration). The results of these experiments suggest that the multimodal integration property of PCs may indeed be conserved from early vertebrates to mammals. Second, a hallmark of mammalian PCs is their representational flexibility (that is, the ability to remap) in response to complete environmental change or disorientation^13,39–41^. We found similar remapping of zebrafish PCs following a complete change in the visual environment coupled with interrupted path integration, which supports the view that PCs form a flexible memory or prediction system.

To fully determine the extent to which zebrafish PCs are functionally analogous to mammalian PCs will require extensive future research. We have primarily focused on exposure to an initially new environment, and future studies will be needed to investigate the storage capacity and stability of the PC network across greater spatial and temporal scales (for example, multiple environments, developmental stages and experience-dependence changes across days and weeks). In addition, the PFs of zebrafish PCs are not completely uniformly distributed throughout the environment but can form clusters, and PCs within a given cluster show weakly correlated activity across environments. This may support recent work in rodents suggesting that spatial tuning across the hippocampus is not entirely independent but may form correlated assemblies^30,31,40,45^. Future studies combining behaviour, functional imaging and synaptic connectivity will be needed to understand the function and neural basis of these weakly correlated ensembles.

Lastly, BVCs, but not PCs, have been identified in the goldfish telencephalon^20^. By contrast, cells that truly fit the mammalian BVC model constitute a small percentage of the zebrafish telencephalon. Although a significant number of larval zebrafish PCs have spatial tuning near boundaries, they may be more functionally analogous to the high density of PCs observed near boundaries in juvenile rodents^46,47^. The differences between zebrafish and goldfish findings could have arisen from several sources. First, the goldfish telencephalon has not been comprehensively recorded. Second, it is not yet entirely conclusive that all goldfish neurons with border activity are functionally closer to BVCs than PCs, because some PCs can also exhibit PF duplications in parallel compartments^48–50^. Finally, it is possible that different fish species indeed rely on different spatial cells for navigation. In the future, more comprehensive recordings and remapping experiments across a variety of fish and other non-mammalian species will be needed to answer these outstanding questions.

## Methods

### Experimental set-up

#### Simultaneous behavioural and neural imaging by tracking microscopy

Tracking microscopy was performed as described in our previous study^7^. Motion cancellation was performed by a custom three-axis motorized system, to be described elsewhere (Mohan et al., manuscript in preparation). To enable animal tracking, the behavioural chamber is illuminated by four custom light strips consisting of narrow-angle, 850 nm infrared light-emitting diodes (LEDs; no. SFH4655-Z, Osram) that deliver infrared light to the chamber by total internal reflection. Ambient white-light illumination in the behavioural chamber is provided by an array of wide-angle white LEDs (no. GW PSLM31.FM).

For recording of neural activity, we used DIFF microscopy to image a brain area of 1,013 × 764 × 150 µm^3^ at cellular resolution, a volume rate of 2 Hz and frame rate of 200 Hz, as described in our previous study^7^. The performance and characteristics of the imaging system have been characterized in depth in our previous study^7^.

#### Chamber construction and experiment design

Several constructions for the chamber wall were used depending on the complexity of the experiment. The top and bottom of all behavioural chambers are made of glass to allow optical access from above and below^7^. Between the glass plates, 1-mm-thick, gas-permeable PDMS walls (Sylgard 184, Dow Corning) were used to create a watertight rigid body as described previously^7^. PDMS chambers were cut using a computer-controlled blade and finished by manual cutting. The chamber was filled with E3 water before fish loading (‘Fish loading, removal and reloading’). Following fish loading we gently closed the chamber by sliding on a top coverslip. Excess fluid was removed to create a watertight seal between the PDMS and the two glass surfaces. Each imaging session lasted 45–100 min to ensure sufficient spatial coverage of the arena (each experiment is described in more detail below). We did not include any fish in our analysis that was quiescent for more than 20 min during the experiment. If not specified explicitly, all chambers were initially new to the fish.

For whole-chamber-rotation experiments (Fig. 4m) and rectangle-to-circle experiments (Fig. 6a), the outer wall (1 mm thick) is made of translucent white PDMS cut into the desired chamber shape. Landmarks are constructed from black PDMS pieces embedded into the outer wall. A clear PDMS inner wall (0.5 mm thick, 1.5 mm wide) placed alongside the outer wall prevents the fish from direct interaction with the outer wall or landmarks. For chamber-rotation experiments, animals were first imaged for 90 min in the rectangular chamber (S1) and then the entire chamber was slowly rotated over 10–20 s as a rigid body without removing the animal (that is, the chamber walls, ceiling, floor and fish all rotated together). Animals were then imaged for a further 60 min (S2).

For experiments that required rotation of the chamber wall without removal of the animal or disassembly of the chamber (Fig. 5m), an outer PDMS wall with a central square cut-out of 60 × 60 mm^2^ was used only for water-sealing the chamber. An inner chamber wall (of distinct size and shape from the outer PDMS wall) was constructed from laser-etched plastic with embedded stainless steel pieces (grade 430), which enabled remote repositioning of the plastic chamber wall using small magnets (3–5 mm in diameter) below the bottom glass of the chamber. Landmarks (when used) were painted onto the wall of the plastic chamber. The plastic chamber wall was placed inside the square cut-out of the outer PDMS wall. Animal movement was restricted to within the plastic chamber walls. Given the flexibility of this chamber design, we also used it for the experiments shown in Fig. 5a,e,i and Extended Data Fig. 9a.

In the wall-rotation experiments we first recorded the animal for 45–90 min (S1) and then the entire chamber assembly was removed from the microscope. Using small magnets underneath the bottom glass of the chamber, we then carefully rotated the chamber walls by 180° before replacing the chamber assembly on the microscope. During this manipulation only the walls rotate but not the fish. Following wall rotation the animal was recorded for a further 45–75 min (S2).

For wall-morphing experiments (Fig. 4i) we constructed a morph chamber consisting of eight pieces of stainless steel (12 × 2 × 1 mm^3^) linked by a flexible ring of silicone (1 mm wide and 0.5 mm tall). The stainless steel pieces can be remotely repositioned using small magnets below the bottom glass of the chamber (as described above), allowing flexible and gradual morphing of the chamber wall. As described above, an outer PDMS wall with a central square cut-out of 60 × 60 mm^2^ was used only for water-sealing the chamber, and the morph chamber was placed inside the square cut-out. Animal movement was restricted to within the morph chamber. We first arranged the morph chamber into a radially near-symmetric octagon and recorded the animal for 45–65 min (S1). Using small magnets underneath the bottom glass of the chamber, we then morphed the chamber wall into an ellipse. During this manipulation we ensured that the walls did not make strong physical contact with the fish. Following morphing, the animal was recorded for a further 45–70 min (S2).

For border-insertion and -removal experiments (Extended Data Fig. 3b) we used a PDMS chamber with an 84 × 15 mm^2^ central cut-out in which the animal was free to move. At the midpoint of the long axis of the chamber we created a hidden rectangular pocket (21.5 × 2 mm^2^) containing a stainless steel rectangular bar (20 × 1.5 × 0.5 mm^3^). Using a small magnet, the stainless steel bar can either be inserted into the chamber (creating an extra border wall) or hidden from the chamber. In S1 (45–95 min) the movable wall was hidden from the animal. Following S1, the movable wall was inserted into the interior of the chamber and the animal recorded for 45–95 min (S2). Following S2, the movable wall was again retracted and hidden from the animal, and the animal recorded again for 45–90 min (S3).

For the landmark-removal experiment (Fig. 4e) we used a PDMS chamber with a 25 × 25 mm^2^ central cut-out. On one edge of the chamber we created two additional PDMS pockets (100 × 2.5 mm^2^) that each contained a 70 × 2 × 0.5 mm^3^ white (acrylic painted), stainless steel, rectangular bar. Landmarks (vertical black stripes) were painted on a portion of one of the stainless steel bars (Fig. 4e) whereas the opposing stainless bar was completely white. In S1 (75–90 min), landmarks were positioned so as to be visible to the fish. Following S1, by applying small magnets to the bottom glass of the chamber, the stainless steel piece with landmarks was remotely moved into the PDMS pocket, effectively hiding the landmarks from the animal within the chamber; the opposing stainless steel bar was not moved. The animal was then recorded again for 60–100 min (S2).

#### Fish loading, removal and reloading

For all experiments, fish were loaded into the chamber using a glass or plastic pipette. For experiments with fish removal between S1 and S2 (Figs. 5a,e,i and 6a) we used the following procedure. Following the first 60–90 min recording (S1) the chamber was removed from the microscope. The top coverslip of the chamber was loosened and then either partially or completely removed. The fish was then removed from the chamber with a glass or plastic pipette. In between sessions, the chamber was thoroughly cleaned with soap and isopropanol and then pressure-rinsed for 2–5 min. For two fish (Extended Data Fig. 8g) we rotated the bottom glass relative to the chamber walls without cleaning, to scramble any potential olfactory cues. We reloaded the fish into the chamber at a different heading and position from those at which the animal was removed. Following fish loading, the chamber was again sealed with its top glass coverslip and transferred to the microscope for a second imaging session (45–100 min). For the ABA experiment (Extended Data Fig. 9a) the same procedure was carried out to load the fish into the B chamber for S2, and to subsequently load the fish into the A chamber for S3.

#### Lights-on/off experiment

To study how PFs are influenced by the luminance of the environment we designed a light/dark protocol. Following 60 min of recording fish exploring the circular PDMS chamber we turned off the white LEDs, providing visible illumination to the chamber. Turning off the white LEDs resulted in a luminance change from 7.05 to under 0.01 µW mm^−2^, measured at the centre of the chamber. To ensure that the transition to lights off did not generate an aversive response, luminance was changed gradually over 1 min. Recording was then resumed for an additional 60 min.

We note that a small blue spotlight with a radius of 0.5 mm is always centred on the brain of the fish under all conditions, due to its requirement for neural imaging. We measured the light scattering from this spotlight to be 0.14 µW mm^−^^2^ within 5 mm of the source.

Light intensity measurements were performed using a photodiode-based optical power sensor with a known detector area (no. S130C, Thorlabs).

### Image registration

#### High-resolution offline registration of fluorescent brain volumes for each animal

Each fluorescent image from the tracking microscope was registered to a high-resolution reference brain volume collected from the same animal. An in-depth description and characterization of the registration pipeline was published in our previous study^7^. Briefly, an initial coarse registration is obtained by optimization of a three-dimensional rigid transformation that maps the moving image to a (possibly tilted) plane within the reference brain volume. This planar surface is then finely subdivided into a deformable surface that is locally adjusted within the reference volume using a regularized piecewise affine transform.

#### Registration to a common reference brain across animals

We use the Computational Morphometry Toolkit (CMTK)^51^ to register each animal to a common reference fish. An atlas fish^52^ was selected to serve as the common reference brain. Each brain was registered onto the reference brain in a series of steps: initialization, rigid, full affine, warp. The coordinate transformation for each individual cell was then saved. The command line commands are listed below:

cmtk make_initial_affine --centers-of-mass moving_image fixed_image initial.list

cmtk registration --initial initial.list --nmi --dofs 6 --dofs 12 --nmi --exploration 8 --accuracy 0.8 -o affine.list moving_image fixed_image

cmtk warp --nmi --threads 160 --jacobian-weight 0 --fast -e 18 --grid-spacing 100 --energy-weight 1e-1 --refine 4 --coarsest 10 --ic-weight 0 --output-intermediate --accuracy 0.5 -o warp.list affine.list

cmtk reformatx --pad-out 0 -o out_image --floating fixed_image moving_image warp.list

cmtk streamxform warp.list <cell_coordinates.txt > cell_coordinates_registered.txt

### Image analysis

#### Extraction of neural activity by NMF

Non-negative matrix factorization (NMF) separates each cell into two components—a spatial footprint and a time-varying activity component—as described in our previous study^27^. Briefly, following registration, we applied constrained NMF^32^ to our whole-brain datasets with nuclear localized fluorescence. NMF was performed for each axial section of a given brain volume. Our axial sections of the reference volume are separated by 2 µm and the average diameter of a zebrafish cell is approximately 5 µm; thus there can be double counting of centroids if a cell spans more than one axial plane. Cell centroids were detected throughout the entire reference brain volume but were included in downstream analysis only if they were sampled in at least 30% of time points throughout the imaging session. We merged these cell centroids belonging to the same cell based on close spatial proximity (horizontal distance 1.4 µm or less, vertical distance 2 µm or less) and highly correlated activity (over 0.7). Following merging, the number of cells was 73,621 ± 10,558 (mean ± s.d.), decreasing by 28.4 ± 2.6% (mean ± s.d.) across animals (*n* = 6 fish).

The fluorescence baseline for each merged cell was estimated using the tenth percentile within a 30 min sliding window. Baseline-corrected fluorescent traces Δ*F*(*t*) were then obtained by subtraction of the estimated baseline from raw fluorescent traces.

#### Identification of PCs

For identification of PCs we first generate a spatial activity map for each neuron (see below), use this map to compute spatial information and specificity^8^ (see below), generate shuffled data by circular permutation to obtain a null distribution for spatial specificity (see below) and, finally, compare the spatial specificity of each cell to the null distribution from shuffled activity and to the distribution of spatial specificity across the brain (see below). The spatial information/specificity criteria for PC identification were originally defined by Skaggs et al.^8^. The criteria we use are similar to those in the existing literature in both mammals and birds, for both electrophysiological and calcium imaging data^16,53^.

A spatial activity map was generated for each neuron, representing the mean neural activity at each spatial location. To calculate the spatial activity map the chamber was divided into square bins (side length 1.2 mm) and then the summed neural activity and occupancy time were calculated for each bin. This resulted in two matrices: a summed neural activity matrix and an occupancy matrix (matrix entries correspond to spatial bins). We then applied a boundary-constrained Gaussian filter (standard deviation one bin, with the boundary defined by the chamber boundary) to these two matrices. The spatial activity map was calculated by dividing the filtered summed neural activity matrix by the filtered occupancy matrix to obtain the filtered average activity in each spatial bin. When the fish was stationary (speed below 0.1 mm s^−1^), the corresponding frames were not included in the calculation of the spatial activity map. For all experimental spatial activity maps (for example, for comparison of spatial maps across sessions) we exclude the first 15 min following initial exposure to the environment in S1. For within-session control (comparison between the first and second halves of S1) we separately generated spatial activity maps for the first and second halves of S1. The first 15 min were not excluded in the within-session control, to ensure sufficient coverage of the environment by the fish trajectory.

From the spatial activity map of each cell, spatial information can be used to quantify how much information is contained by that cell about the location of the animal, in units of bits per second^8^. For each cell, spatial information *I* was calculated as

$$I=\sum _{x}\lambda \left(x\right){{\rm{lo}}g}_{2}\frac{\lambda \left(x\right)}{\lambda }P(x),$$

where *x* is a spatial bin, *P*(*x*) is the probability that the fish is in spatial bin *x*, *λ*(*x*) is the mean activity of the cell when the fish is in spatial bin *x* and *λ* is average neural activity, computed as $\lambda ={\sum }_{x}\lambda (x)P(x)$.

Based on the equation above, cells with high average neural activity tend to have higher spatial information. To normalize for this we calculate specificity *s* as

$$s=\frac{I}{\lambda }.$$

In other words, specificity is spatial information divided by average neural activity, resulting in units of bits per activity unit.

Due to our baseline correction, bins occasionally have negative average activities. Such bins, as well as those with less than 1 s total occupancy time following Gaussian filtering, were not included in the calculation of spatial information and specificity.

To test the significance of the specificity of each cell we use circular permutation to construct a null distribution. For each cell we define the set of valid timepoints as the frames in which neural activity was recorded and fish movement speed was above 0.1 mm s^−1^. A null distribution for specificity is then estimated by measurement of specificity after circularly permuting the neural activity vector within the valid time points by 1,000 offsets (each offset is 0.5 s, so it covers from −250 to +250 s). The specificity of a given cell is converted to a specificity *z*-score by subtracting the mean specificity of the null distribution and dividing by the standard deviation of the null distribution. To test the significance of the specificity of a cell at the population level, a population specificity *z*-score is also calculated by subtracting the mean specificity of all recorded cells and dividing by the standard deviation of the specificity of all recorded cells.

To be classified as a place-encoding cell, a cell is required to have a specificity *z*-score larger than or equal to 5, a population specificity *z*-score larger than or equal to 3 and a specificity value of over 0.01 bits per activity unit.

#### Defining place fields

The place field (PF, or firing field) of each neuron is defined as the set of spatial bins with activity above 80% of peak activity (with peak defined as the 95th percentile) of the spatial activity map. A more in-depth and systematic analysis of unimodal and multimodal PFs is described in Extended Data Fig. 1i–k, in which the activity threshold was swept from 50 to 80%. The location of the PF is represented by its COM for cells with a single PF. For cells with multiple PFs (as distinct components in the map) we use the COM of the component (over 20 bins, to avoid spurious PFs due to noise), with the highest peak activity (defined as the 95th percentile of the component) as the location of its primary PF. Only PCs with a PF size of less than 30% of chamber size are included in maps of the distribution of PFs and in the analysis of PF shift. PFs are used only for analysis of PF shift and visualization of PF location across an environment. All other analyses of PC activity, such as PF correlation, PV correlation, change in specificity and positional decoding, use the spatial activity map directly.

#### Rigid and non-rigid registration of spatial activity maps across sessions

Experiments were conducted with various chambers, sometimes in varying orientations, sometimes before and after morphing the chamber into different shapes. For comparison of spatial activity maps across these chambers we developed methods to register maps across sessions. When there was no change in chamber wall geometry (for example, whole-chamber rotation), registration was performed by rotation and translation of the S2 activity map (that is, a rigid transformation) so that activity in both sessions was represented in terms of the spatial bins of S1, thus facilitating comparisons (‘PV correlation, PF correlation and PF shift’).

Otherwise we performed non-rigid transformation to register spatial activity maps across sessions. Our strategy was first to establish a correspondence between the chamber walls of both sessions, then to map each spatial bin of S2 to a set of spatial bins in S1 according to their distance to the wall anchor points, and finally to represent the activity of S2 in terms of the spatial bins of S1. Each of these steps is described in greater detail below.

First we detected the walls of the chambers in both sessions and defined a set of anchor points on the chamber walls. Because the precise number of detected wall points could differ between sessions, we used linear interpolation to upsample the wall points of the session with fewer points, such that the number of anchor points was the same across sessions. The correspondence between anchor points across sessions was established based on either landmarks (for example, the wall-morphing experiment with fish removal; Fig. 5i) or the polar angle of the wall within the microscope reference frame in experiments with no clear match between geometry or landmarks (for example, rectangle-to-circle remapping (Fig. 6a) and ABA remapping (Extended Data Fig. 9a)), or by systematic consideration of every rotation of the anchor points in S2 relative to S1 (Extended Data Figs. 8c,d and 10; ‘Non-rigid transformation with the best rotation angle’).

Next, for each spatial bin centre (*x*,*y*) in S2 we compute a corresponding location $({x}^{{\prime} }\,,{y}^{{\prime} })$ in S1 using a COM procedure. Specifically, for each anchor point $({x}_{a},{y}_{a})$ in S2 we associate a weight $1/{d}^{2}$, where *d* is the distance between the anchor point $({x}_{a},{y}_{a})$ and the spatial bin centre (*x*,*y*) in S2. We then use the previously established wall correspondence between both sessions to transfer these weights to the anchor points in S1. The corresponding location $({x}^{{\prime} }\,,{y}^{{\prime} })$ in S1 is then defined as the COM of the S1 anchor points (that is, the weighted sum of the S1 anchor points divided by the sum of the weights).

Finally we represent the activity of S2 in terms of the spatial bins of S1. The computed location $({x}^{{\prime} }\,,{y}^{{\prime} })$ in S1 is generally between the spatial bin centres of the S1 activity map, so we identify the 4 × 4 spatial bins with bin centres (*x*,*y*) with ${\rm{floor}}({x}^{{\prime} })-1\le x\le {\rm{ceil}}\,({x}^{{\prime} })+1$ and ${\rm{floor}}(\,y)-1\le y\le {\rm{ceil}}\,(\,y{\prime} )+1$. In this way we associate each S2 spatial bin with 4 × 4 spatial bins in S1. This procedure ensures that each S1 spatial bin is associated with at least one S2 bin. We then average the activity of all S2 bins that are associated with a given spatial bin in S1, yielding a representation of the activity of S2 in terms of the spatial bins of S1.

#### Non-rigid transformation with the best rotation angle

Non-rigid transformation of the S2 maps described above is generally applied assuming a rotation angle of 0° between sessions (for example, Figs. 4i, 5i and 6a). For systematic investigation of whether a coherent map rotation had occurred between sessions (Extended Data Fig. 8d), 72 incremental rotations (covering 360°) were applied to the S2 anchor points followed by non-rigid registration as described above. The best rotation angle is identified by the maximum PF correlation (‘PV correlation, PF correlation and PF shift’) across sessions. We refer to this procedure as ‘non-rigid transformation with the best rotation angle’. Note that this is also done for the early–late control in Extended Data Fig. 8c,e.

To test whether the improvement in PF correlation, from ‘non-rigid registration assuming an angle of 0°’ to ‘non-rigid transformation with the best rotation angle’, is significant, we shuffle the cell identity in S2. Post shuffle, we then compare the improvement in PF correlation from non-rigid registration assuming an angle of 0° with non-rigid transformation with the best rotation angle. This was repeated 1,000 times to generate a null distribution. The *P* value in Extended Data Fig. 10 is calculated by counting the percentage of shuffles in which the real data improve by less than the shuffle control.

#### PV correlation, PF correlation and PF shift

For comparison of population-level activity across sessions, a population vector correlation (that is, PV correlation) was computed for each spatial bin that is shared across sessions. For each neuron we first compute a Δ*F*/*F* spatial activity map for each session. To do this we estimate a baseline fluorescent signal for each map by taking the mean of spatial bins with fluorescent signal below the 20th percentile. The Δ*F*/*F* of each spatial bin is then computed as follows:

$$\frac{A-{\rm{baseline}}}{{\rm{baseline}}+c}$$

where *A* is the mean fluorescent signal for a spatial bin. A pseudocount *c* is added when the baseline is below 10. For each spatial bin we obtained two vectors of population activity (one for each session). The length of the vector is equal to the total number of telencephalic PCs identified from either session. Correlation between the two activity vectors yields the PV correlation for a given spatial bin.

To determine the similarity of spatial activity maps we computed the correlation between spatial activity maps (that is, PF correlation or spatial correlation). Correlation was performed on spatial bins that are shared across both maps and normalized by the mean and variance of each map. All telencephalic cells identified as PCs in either session were used.

To measure the extent of PF shift across sessions, for each neuron we identified the COM of its PF in each session. We define PF shift as the distance between the COM of the cell’s PF in S1 and S2. Only cells with confined PFs (firing field size less than 30% of chamber size; section ‘Defining place fields’) in both sessions are included for this analysis.

For the within-session control we generate two separate spatial activity maps corresponding to the early and late halves of S1. PV correlation, PF correlation and PF shift are computed from the within-session control spatial activity maps using the procedure described above. For comparison with control we ensure that the same set of neurons (for PV correlation, PF correlation and PF shift) and spatial bins (for PV correlation and PF correlation) are used. One-sided Wilcoxon signed-rank tests are used to measure whether PF correlation and PV correlation are significantly lower than control, and whether PF shift is significantly higher than control.

#### Blurring of the spatial activity map

Blurring is the broadening of a PF at a given point on its boundary in the radially outward direction. For estimation of this blurring effect of PFs due to the speed of the fish, combined with calcium indicator dynamics, we compute an upper and a lower bound. The lower bound is computed by assuming a 10 Hz firing rate and a speed of the mean minus standard deviation; the upper bound assumes a firing rate of 100 Hz with a speed of the mean plus standard deviation. Speed distribution consists of pooled speed data from seven fish (Extended Data Fig. 1f). The data for the half-decay times for different firing rates are taken from ref. ^54^. The half-time of temporal fluorescence decay is multiplied by the speed value to obtain the half-distance for spatial fluorescence decay (Extended Data Fig. 1g). Of this exponential spatial decay, the distance needed for a decay down to 80% of starting value is computed (Extended Data Fig. 1h); this matches our definition of PF and is used as a blurring estimate. Based on the behavioural data (Extended Data Fig. 1f) and calcium indicator kinetics, which both depend on firing rate^54^, we estimate the radius of the PFs to be spatially blurred by 0.17–1.31 mm.

#### Isomap and quantification

Isomap^37^ embedding was performed using Scikit-learn^55^ with ‘n_neighbors = 100’. A rectangular matrix representing the population activity of all telencephalic PCs in a span of 30 min was constructed, an Isomap manifold was fit to the data (with each point in the manifold representing the population activity at one time point) and finally a two-dimensional embedding was extracted for visualization in Fig. 3a and computation in Fig. 3b. Because the implementation does not handle missing data, we filled in missing values in the activity trace for individual cells with the nearest preceding available value. In the case of missing values at the beginning of the experiment, a backwards filling is applied. For Fig. 3a,b the early (0–30 min) and late (60–90 min) windows of population activity data were fit separately and therefore have different two-dimensional embeddings. For Supplementary Video 2 we fit an Isomap manifold to the final 30 min of imaging S1 (before chamber rotation; Fig. 4m) to establish stable axes for two-dimensional embedding, and then repeatedly transformed each window of 30 min of population activity data into this embedding. Standard exclusion criteria based on movement were applied (spatial activity map in ‘Identification of PCs’).

To quantify the relationship between the two-dimensional manifold and the physical position of the fish we use neighbour distance, which quantifies the degree to which local neighbours in the manifold space are also local neighbours in the physical space of the chamber. In a given 30 min time window we analyse each time point in the two-dimensional manifold space (the ‘seed point’) by selecting its 30 nearest neighbours in the manifold space, then measuring the physical distance in the chamber between those 30 points and the seed point and averaging to obtain the mean physical neighbour distance of the seed point. We then compute the overall average physical neighbour distance by averaging this across all time points in the manifold. As a baseline, for each seed point, 30 random neighbours in the manifold are chosen and then physical neighbour distance is computed as before.

For analyses comparing between two sessions, or between the early and late intervals of a session (Fig. 3), the occupancy of each spatial bin was equalized by subsampling. That is, for each spatial bin, we identified the session or time interval having a lesser number of time points and randomly subsampled from the session or time window with more time points, so that both time windows had the same number of time points in each spatial bin.

#### Direct basis decoder

The direct basis decoder^36^ is a linear decoder with no free parameters that predicts the animal’s position at each individual time point by a linear combination of the spatial activity maps, weighted with the activity of the corresponding place-encoding cell. All decoding and map construction was performed only at those time points at which the fish was moving (fish speed greater than 0.1 mm s^−1^). Even though no parameters had to be learned, it is still necessary to compute the spatial activity map of each cell. To avoid circularity between computing the spatial activity map and testing the decoder we used a cross-validation scheme in which the neural data were divided into non-overlapping 1 min chunks. To test the decoder on each 1 min chunk we first computed the spatial activity map without including the test chunk or its two neighbouring chunks. The predictions of all 1 min chunks were concatenated as the prediction of the whole dataset. Decoder error was then computed as the mean distance between the predicted and actual position of the fish across the dataset.

Spatial activity maps were constructed as described above using spatial bins (side length 1.2 mm) and boundary-constrained Gaussian smoothing. Apart from the analysis shown in Extended Data Fig. 5c, the activity used in this map construction is time shifted by 2 s relative to the location of the fish, to counteract potential calcium lag dynamics. All maps were standardized by mean and standard deviation. To prepare the decoder, a 7.5 s boxcar average filter was applied to the activity and then maps of the most active 30% of cells were weighted by their respective activity and summed to form the decoder map. This nonlinear thresholding step was designed to omit low-intensity signals that would contribute noise to the decoding. The decoder map was then normalized for density differences in representation. To this end we calculated a representation histogram across all spatial bins, defined for each spatial bin as the number of PCs containing that bin in their primary PF (Defining place fields). Because we found that the effect of representation density on decoding error was sublinear, we normalized the decoder map by the third root of the representation histogram. The position estimate for each time point was then calculated by the COM of the 99th percentile of the decoder map. If not specified differently, 1,000 telencephalic cells of the highest spatial tuning per animal were used for all decoding analyses. For selection of the top spatially tuned cells we ranked each telencephalic place-encoding cell according to its specificity *z*-score and also according to its population specificity *z*-score, and then assigned its final rank according to the worse of the two ranks (Fig. 2f). This allows for a fair comparison between fish that may have varying numbers of PCs (Fig. 1g). For selection of the minimum number of cells needed for good decoding, an iterative, greedy algorithm was used (Fig. 2g). Starting with zero neurons, we find the single best neuron to add to the decoding set to minimize the resulting decoder error and then iteratively repeat this same greedy selection procedure to grow the decoding set, one neuron at a time.

For the analysis of decoder error by brain region (Fig. 2e), the 1,000 top spatially tuned cells were chosen for each defined region (whole brain, telencephalon, mes- and rhombencephalon). The random cell population was randomly selected from cells with a population *z*-score less than 1, to explicitly contain no PCs. We defined two baselines for evaluation of the decoder performance. For the uniform random baseline we measured the average decoder error of a decoder that outputs random positions sampled uniformly from the chamber. This procedure was repeated 1,000 times, with the mean decoder error taken as the uniform random baseline. For the behaviour-informed baseline we measured the average decoder error of a decoder that outputs the COM of fish positions in the chamber.

For all analyses, if not specified differently, only the final 75 min of each experiment were used for decoding. In the analysis of the decoder error as a function of number of cells included (Fig. 2f), the threshold for spatial tuning was gradually lowered until 10,000 cells were obtained (*n* = 7 fish). The two fish with fewer than 10,000 recorded cells in the telencephalon were hence excluded from this analysis. To avoid circularity, for Fig. 3d the shuffled specificity *z*-score and population *z*-score were calculated separately for the first and last 30 min of the experiments. Decoding for these two time windows was therefore based on the respective top-ranked PCs within each window.

We find that the non-redundant decoder starts to outperform the decoder using all cells from 17 cells onwards. The worse performance of the decoder using all cells is a consequence of the simple linear decoder design, which values the information of all cells equally; hence, less specific neurons can influence the decoder and slightly worsen the results. The advantages of this decoder, however, are the limited assumptions of the model, easy interpretability and impossibility of overfitting.

#### Regression and prediction of neural activity

We used ordinary least-squares regression to measure how much of the variance in place-encoding cell activity can be explained by different behavioural variables, including physical location, heading and speed. This is a much simpler model compared with previous work using a generalized linear model^56^. The behaviour variables were discretized into bins, and an indicator function for each bin was added as a regressor in the model. The regression model can be summarized by the following formula:

$${\lambda }_{n}\left(t\right)={\mu }_{n}+\mathop{\sum }\limits_{i=1}^{L}{w}_{i,n}^{\left(x\right)}{x}_{i,t}+\mathop{\sum }\limits_{j=1}^{H}{w}_{j,n}^{\left(h\right)}{h}_{j,t}+\mathop{\sum }\limits_{k=1}^{S}{w}_{k,n}^{\left(s\right)}{s}_{k,t}$$

where ${\lambda }_{n}\left(t\right)$ is the baseline-corrected neural activity for cell *n* at time *t*, ${\mu }_{n}$ represents baseline activity, ${w}_{i,n}^{(x)}{x}_{i,t}$ represents the contribution from the physical location of the fish, ${x}_{i,t}$ is the activity for spatial bin *i* at time *t*, *L* is the number of spatial bins (15 × 8 = 120), ${w}_{j,n}^{\left(h\right)}{h}_{j,t}$ represents the contribution from the heading of the fish, ${h}_{j,t}$ is the activity for heading bin *j* at time *t*, *H* is the number of heading bins (24), ${w}_{k,n}^{(s)}{s}_{k,t}$ represents the contribution from the speed of the fish, ${s}_{k,t}$ is the activity for speed bin *k* at time *t* and *S* = 24 is the number of speed bins. For comparison of contributions from spatial location, heading and speed, both individually and together, we tested ordinary least-squares regression in four cases: ${\lambda }_{n}(t)={\mu }_{n}+{\sum }_{i=1}^{L}{w}_{i,n}^{(x)}{x}_{i,t}$ (that is, with only spatial indicator functions), ${\lambda }_{n}(t)={\mu }_{n}+{\sum }_{j=1}^{H}{w}_{j,n}^{(h)}{h}_{j,t}$ (that is, with only heading indicator functions), ${\lambda }_{n}(t)={\mu }_{n}+{\sum }_{k=1}^{S}{w}_{k,n}^{(s)}{s}_{k,t}$ (that is, with only speed indicator functions) and, finally, the complete equation shown above. All models included a weak L2 regularization on the parameters with a penalty coefficient of 1^−10^. For each regression model we report the resulting distribution of *R*^2^ values across the telencephalic PCs shown in Extended Data Fig. 1b. Similarly, the distribution of *R*^2^ values across other telencephalic cells is shown in Extended Data Fig. 1c. Standard exclusion criteria based on movement speed were applied (see spatial activity map in ‘Identification of PCs’).

#### Whole-brain PC maps

Following projection of the location of PCs from all fish to the same reference fish^52^, we accumulated all PCs into a three-dimensional histogram representing how many PCs were detected in each three-dimensional bin within the reference volume. We then convolved the three-dimensional histogram with a spherical convolution mask of radius 5 µm, such that each place-encoding cell contributes an increment of one count to all bins within a 5 µm radius. Maximum-intensity projections were then computed to visualize the anatomical distribution from multiple views (Fig. 1f).

#### BVC model

To search for cells whose firing properties follow the BVC model^34^ we fitted a BVC model (see below) to candidate neurons in the telencephalon. The model predicts that the activity of these cells, *f*, integrates each boundary point (represented in polar coordinates *r* and *θ* relative to the animal) according to

$${\rm{\delta }}f=g(r,\theta ){\rm{\delta }}\theta ,$$

where $g(r,\theta )$ represents the firing rate relative to a preferred firing orientation *ϕ* and distance *d* from the boundary:

$$g(r,\theta )=A\frac{\exp \left[\frac{{-(r-d)}^{2}}{2{\sigma }_{{\rm{rad}}}^{2}}\right]}{\sqrt{2{\rm{\pi }}{\sigma }_{{\rm{rad}}}^{2}}}\frac{\exp \left[\frac{{-(\theta -\phi )}^{2}}{2{\sigma }_{{\rm{ang}}}^{2}}\right]}{\sqrt{2{\rm{\pi }}{\sigma }_{{\rm{ang}}}^{2}}}+c.$$

Here, ${\sigma }_{{\rm{rad}}}$ and ${\sigma }_{{\rm{ang}}}$ are the width of radial and angular tuning, respectively. Compared with the original model^34^, which treats ${\sigma }_{{\rm{rad}}}$ as a variable that linearly depends on *d*, we treat ${\sigma }_{{\rm{rad}}}$ as a constant to be fit. *A* is a scaling factor and *c* is the baseline. Note that the bin size for spatial activity maps for this experiment is slightly smaller (side length 1.1 mm), to ensure sufficient resolution for accurate detection and representation of the inserted wall. When fitting the model to spatial activity maps, we first normalize the map by its standard deviation. During the fitting we constrain *d* to be between 0 and 10 bins (assuming BVCs fire close to the boundary), *ϕ* between −π and π, both ${\sigma }_{{\rm{rad}}}$ and ${\sigma }_{{\rm{ang}}}$ between 0 and 10, *A* between 0 and 100 and *c* between −10 and +10 activity units.

To test whether the activity of a given neuron is predicted by the BVC model we performed a three-session experiment—after a baseline session (45–95 min), an interior wall was introduced for S2 (45–95 min) and removed for S3 (45–95 min) (Extended Data Fig. 3b). We first identified all cells passing a minimum specificity criterion (over 0.01 bits per activity unit for all three sessions). Given the orientation of the inserted wall, only cells with PFs parallel to the inserted wall (from −45 to 45° or 135 to 225° in both S1 and S3) are suitable candidates for BVC analyses. We then fit the BVC model to the spatial activity maps of these candidate cells in S1 and then used the fitted model to generate predicted activity maps across sessions (with or without wall insertion).

We computed the Pearson correlation coefficient between the observed spatial activity map of each session with the predicted activity map generated by the fitted model. To test the significance of the Pearson correlation coefficient we circularly permuted the observed spatial activity maps approximately 1,100 times (same as the number of bins in the map) and recalculated the Pearson correlation coefficient for each permutation to construct the null distribution for a one-sided shuffle test.

Crucially, the BVC model predicts a duplication of PFs in S2. This duplicated firing field is expected to be absent in S1 and S3, which are predicted to have the same spatial activity map. Thus, to be classified as a BVC, a cell has to be consistent with the BVC model in all three sessions:

1. For S1, Pearson correlation between the observed spatial activity map and model-predicted activity should be significantly higher relative to shuffle (*P* < 0.05, one-sided shuffle test as described above).
2. For S2, Pearson correlation between the observed spatial activity map and model-predicted activity should be significantly higher relative to shuffle (*P* < 0.05, one-sided shuffle test) and the observed spatial activity map should have higher Pearson correlation with model-predicted activity with wall insertion than model-predicted activity without wall insertion.
3. For S3, the spatial activity map should return to the map observed in S1 (PF correlation between S1 and S3 should be significantly higher relative to shuffle; *P* < 0.05, one-sided shuffle test).

#### Neighbourhood analysis

To quantify the degree to which potential clustering of PFs is maintained across sessions, we performed a neighbourhood analysis for each neuron (Fig. 6e). First, for each neuron we ranked all other neurons by their PF correlation to the neuron of interest in S1. We define neurons with the highest PF correlation as neighbours, with a systematically varied inclusion threshold from top 2% to top 100%. We define neighbour retention % as the number of neurons that remain neighbours in S2 divided by the number of original neighbours in S1. The mean neighbour retention % across all telencephalic PCs from either session is then plotted. This analysis is also carried out separately for cells whose PF is close to the edge (nearest distance of COM to edge 3 mm or less) and for cells whose firing field is away from the edge (nearest distance to edge greater than 3 mm). To avoid any potential contribution from imperfect cell merging we restricted this analysis to pairs of cells with a minimum anatomical distance of over 20 µm. A one-sided Wilcoxon signed-rank test was used to quantify whether neighbour retention % in the experiment was significantly worse than a within-session positive control (comparison between the early and late periods of S1). As a negative control we shuffled the cell indices in the second session 1,000 times to randomize relationships between neurons. Post shuffle, we calculated mean neighbour retention % across all telencephalic PCs from either session to generate a null distribution (that is, a shuffle control). A *P* value is calculated by counting the percentage of shuffles in which the mean neighbour retention % is higher than the shuffle control. For these significance tests we fixed the number of top correlated neighbours to 10%.

### Significance tests for sample comparison

Wilcoxon signed-rank tests were used for paired location comparisons with equal sample size. Mann–Whitney *U*-tests were used for unpaired location comparison.

#### Animal care and transgenic lines

Experiments were carried out in accordance with the Animal Welfare Office at the University of Tübingen and the Regierungspräsidium. All experiments in rectangular chambers, and ABA experiments, used Tg(elavl3:H2B-GCaMP6s^+/+^) with nacre (mitfa^−/−^) at 6–9 days postfertilization. The remainder of the experiments used Tg(elavl3:H2B-GCaMP8s^+/+^ or GCaMP8s^+/−^) with nacre (mitfa^−/−^) at 6–13 days postfertilization. In three of the rectangle-to-circle experiments the fish expressed an additional allele of Tg(elavl3:GCaMP6s). Fish were reared on a 14/10 h light/dark cycle at 25 °C. They were maintained in sets of 30 in E3 water and fed dry food daily.

#### Computer hardware for data acquisition and analysis

The tracking microscope was controlled by a rack-mounted, 12-core workstation with a Geforce RTX 3080 Ti graphics processing unit. Offline data analysis was performed using eight rack-mounted, 8- to 16-core Linux servers with four graphics processing units each (Geforce RTX 3080 Ti, RTX 2080 Ti or GTX 1080 Ti), or at the Max Planck Computing and Data Facility (Raven, A100-SXM4).

### Reporting summary

Further information on research design is available in the Nature Portfolio Reporting Summary linked to this article.

## Online content

Any methods, additional references, Nature Portfolio reporting summaries, source data, extended data, supplementary information, acknowledgements, peer review information; details of author contributions and competing interests; and statements of data and code availability are available at 10.1038/s41586-024-07867-2.

## Supplementary information

Supplementary Table 1 *P* values for all statistical tests. For all *P* values of statistical tests, if not mentioned in the figure legend they are provided in the table.

Reporting Summary

Supplementary Video 1 Activity of three PCs over the course of one experiment. Fish trajectory is plotted in grey, and cell activation (Δ*F*(*t*) greater than 3 s.d.) is shown as coloured dots (cyan, magenta and yellow representing the three cells). Activity is smoothed with a 2.5 s boxcar average filter.

Supplementary Video 2 Activity manifold untangling over time. We fit an Isomap manifold to the final 30 min of imaging S1 (before chamber rotation; Fig. 4m) to establish stable axes for two-dimensional embedding, and then repeatedly transformed each window of 30 min of population activity data into this embedding (Methods).

## Source data

Source Data Fig. 1

Source Data Fig. 2

Source Data Fig. 3

Source Data Fig. 4

Source Data Fig. 5

Source Data Fig. 6

## Data Availability

The data that support the findings of this study can be made available from the corresponding authors on request. Source data are provided with this paper.

## Code availability

All analysis software was written in Julia 1.10 and CUDA C using the Julia package ecosystem (Optim.jl, PyPlot.jl, PyCall.jl, Images.jl, Cairo.jl, HDF5.jl, CUDA.jl)^57,58^. Graphics processing was implemented for fish tracking, as well as for online and offline image registration. Data analysis code is available online at https://github.com/RoLiLab/fish_place_cell_Yang_2024.
