# Publisher Correction: A population code for spatial representation in the zebrafish telencephalon

**DOI:** 10.1038/s41586-024-08009-4

**Published:** 2024-09-13

**Authors:** Chuyu Yang, Lorenz Mammen, Byoungsoo Kim, Meng Li, Drew N. Robson, Jennifer M. Li

**Affiliations:** 1 Max Planck Institute for Biological Cybernetics, Tuebingen, Germany; 2 University of Tuebingen, Tuebingen, Germany; 3 Present Address: Department of Neurobiology and Behavior, Cornell University, Ithaca, NY USA; 4 Present Address: Shanghai Institute of Microsystem and Information Technology, Chinese Academy of Sciences, Shanghai, China; 5 Present Address: INSIDE Institute for Biological and Artificial Intelligence, Shanghai, China

**Keywords:** Neural circuits, Navigation

Correction to: *Nature* 10.1038/s41586-024-07867-2 Published online 28 August 2024

In the version of the article initially published, two present addresses for Meng Li (Shanghai Institute of Microsystem and Information Technology, Chinese Academy of Sciences, Shanghai, China and INSIDE Institute for Biological and Artificial Intelligence, Shanghai, China) were missing and have now been added to the HTML and PDF versions of the article.

