# Supplementary material for: A population code for spatial representation in the zebrafish telencephalon
Source: Nature. 2024 Aug 28;634(8033):397–406. doi: 10.1038/s41586-024-07867-2 (PMC11464381; doi:10.1038/s41586-024-07867-2)
Supplement: Supplementary file 1 — P values for all statistical tests. For all P values of statistical tests, if not mentioned in the figure legend they are provided in the table. [file 41586_2024_7867_MOESM1_ESM.docx]

Table 1. p-values (a-y) and sample size (z)

| a. Figure 3b (one-sided Mann-Whitney U test), manifold space / neural space | | | | | | | | | | | | |
| --- | --- | --- | --- | --- | --- | --- | --- | --- | --- | --- | --- | --- |
| Fish 1 | 2.132.E-110 / 1.174.E-17 | | | | | | | | | | | |
| Fish 2 | 4.841.E-25 / 1.555.E-54 | | | | | | | | | | | |
| Fish 3 | 2.702.E-04 / 9.496.E-01 | | | | | | | | | | | |
| Fish 4 | 3.797.E-62 / 9.814.E-144 | | | | | | | | | | | |
| Fish 5 | 2.789.E-114 / 1.179.E-96 | | | | | | | | | | | |
| Fish 6 | 9.927.E-01 / 4.627.E-16 | | | | | | | | | | | |
| Fish 7 | 2.427.E-29 / 8.660.E-24 | | | | | | | | | | | |
| b. Figure 3c (one-sided Mann-Whitney U test) | | | | | | | | | | | | |
| Fish 1 | 5.153.E-17 | | | | | | | | | | | |
| Fish 2 | 2.907.E-04 | | | | | | | | | | | |
| Fish 3 | 1.206.E-05 | | | | | | | | | | | |
| Fish 4 | 5.013.E-18 | | | | | | | | | | | |
| Fish 5 | 2.573.E-03 | | | | | | | | | | | |
| Fish 6 | 3.484.E-03 | | | | | | | | | | | |
| Fish 7 | 2.235.E-03 | | | | | | | | | | | |
| c. Figure 3d (one-sided Mann-Whitney U test) | | | | | | | | | | | | |
| Fish 1 | 5.939.E-01 | | | | | | | | | | | |
| Fish 2 | 4.371.E-33 | | | | | | | | | | | |
| Fish 3 | 5.967.E-01 | | | | | | | | | | | |
| Fish 4 | 1.395.E-50 | | | | | | | | | | | |
| Fish 5 | 3.301.E-117 | | | | | | | | | | | |
| Fish 6 | 3.602.E-09 | | | | | | | | | | | |
| Fish 7 | 2.639.E-20 | | | | | | | | | | | |
| d. Figure 3e (one-sided Wilcoxon signed-rank test) | | | | | | | | | | | | |
| Fish 1 | 1.362.E-44 | | | | | | | | | | | |
| Fish 2 | 9.997.E-01 | | | | | | | | | | | |
| Fish 3 | 2.209.E-09 | | | | | | | | | | | |
| Fish 4 | 1.265.E-22 | | | | | | | | | | | |
| Fish 5 | 4.461.E-09 | | | | | | | | | | | |
| Fish 6 | 1.074.E-15 | | | | | | | | | | | |
| Fish 7 | 7.037.E-04 | | | | | | | | | | | |
| e. Figure 4d (Light / Dark) (one-sided Wilcoxon signed-rank test) | | | | | | | | | | | | |
|  | PF cor. | | | | PV cor. | | | | PF shift | | | |
| Fish 1 | 1.000.E+00 | | | | 1.000.E+00 | | | | 9.700.E-01 | | | |
| Fish 2 | 1.000.E+00 | | | | 3.691.E-02 | | | | 4.628.E-01 | | | |
| Fish 3 | 1.000.E+00 | | | | 2.655.E-13 | | | | 9.722.E-01 | | | |
| f. Figure 4h, Extended Data Figure 6a (Landmark removal) (one-sided Wilcoxon signed-rank test) | | | | | | | | | | | | |
|  | PF cor. | | | | PV cor. | | | | PF shift | | | |
| Fish 1 | 1.456.E-05 | | | | 1.000.E+00 | | | | 2.975.E-06 | | | |
| Fish 2 | 1.000.E+00 | | | | 9.999.E-01 | | | | 2.193.E-01 | | | |
| Fish 3 | 5.415.E-01 | | | | 1.000.E+00 | | | | 6.256.E-01 | | | |
| g. Figure 4l, Extended Data Figure 6a (Wall morphing) (one-sided Wilcoxon signed-rank test) | | | | | | | | | | | | |
|  | PF cor. | | | | PV cor. | | | | PF shift | | | |
| Fish 1 | 6.435.E-01 | | | | 1.211.E-34 | | | | 1.243.E-02 | | | |
| Fish 2 | 1.000.E+00 | | | | 8.081.E-01 | | | | 7.071.E-01 | | | |
| Fish 3 | 7.340.E-53 | | | | 9.236.E-10 | | | | 1.913.E-10 | | | |
| Fish 4 | 6.297.E-01 | | | | 9.527.E-01 | | | | 9.720.E-01 | | | |
| h. Wall morphing original (one-sided Wilcoxon signed-rank test) | | | | | | | | | | | | |
|  | PF cor. | | | | PV cor. | | | | PF shift | | | |
| Fish 1 | 2.816.E-10 | | | | 1.374.E-17 | | | | 2.360.E-03 | | | |
| Fish 2 | 7.792.E-01 | | | | 3.287.E-25 | | | | 3.414.E-05 | | | |
| Fish 3 | 1.056.E-72 | | | | 1.237.E-22 | | | | 1.699.E-18 | | | |
| Fish 4 | 7.554.E-01 | | | | 5.703.E-01 | | | | 3.074.E-08 | | | |
| i. Figure 4p,q, Extended Data Figure 6a (Chamber rotation) (one-sided Wilcoxon signed-rank test) | | | | | | | | | | | | |
|  | PF cor. | | | | PV cor. | | | | PF shift | | | |
| Direct comparsion | | | | | | | | | | | | |
| Fish 1 | 9.970.E-157 | | | | 9.961.E-125 | | | | 3.203.E-38 | | | |
| Fish 2 | 5.448.E-261 | | | | 5.753.E-127 | | | | 1.362.E-89 | | | |
| Fish 3 | 1.583.E-79 | | | | 1.904.E-129 | | | | 9.134.E-30 | | | |
| Rotation corrected | | | | | | | | | | | | |
| Fish 1 | 1.000.E+00 | | | | 1.000.E+00 | | | | 1.000.E+00 | | | |
| Fish 2 | 2.169.E-09 | | | | 9.654.E-27 | | | | 5.418.E-02 | | | |
| Fish 3 | 1.000.E+00 | | | | 1.000.E+00 | | | | 1.000.E+00 | | | |
| j. Figure 5d, Extended Data Figure 6a (Fish removal, No change), Extended Data Figure 8f-g (one-sided Wilcoxon signed-rank test) | | | | | | | | | | | | |
|  | PF cor. | | | | PV cor. | | | | PF shift | | | |
| Fish 1 | 3.251.E-11 | | | | 7.811.E-07 | | | | 1.000.E+00 | | | |
| Fish 2 | 7.716.E-42 | | | | 3.652.E-103 | | | | 2.213.E-02 | | | |
| Fish 3 | 1.000.E+00 | | | | 1.000.E+00 | | | | 8.545.E-01 | | | |
| Fish 4 | 4.150.E-56 | | | | 2.348.E-125 | | | | 1.422.E-07 | | | |
| k. Figure 5h, Extended Data Figure 6a (Landmark removal+ Fish removal) (one-sided Wilcoxon signed-rank test) | | | | | | | | | | | | |
|  | PF cor. | | | | PV cor. | | | | PF shift | | | |
| Fish 1 | 3.393.E-14 | | | | 4.585.E-115 | | | | 1.456.E-01 | | | |
| Fish 2 | 3.256.E-36 | | | | 1.122.E-103 | | | | 2.293.E-01 | | | |
| Fish 3 | 4.361.E-44 | | | | 1.386.E-83 | | | | 6.847.E-04 | | | |
| Fish 4 | 1.185.E-34 | | | | 2.165.E-14 | | | | 1.751.E-09 | | | |
| l. Figure 5l, Extended Data Figure 6a (Wall morphing +Fish removal) (one-sided Wilcoxon signed-rank test) | | | | | | | | | | | | |
|  | PF cor. | | | | PV cor. | | | | PF shift | | | |
| Fish 1 | 2.404.E-02 | | | | 9.136.E-27 | | | | 9.960.E-01 | | | |
| Fish 2 | 4.389.E-28 | | | | 7.359.E-24 | | | | 3.255.E-05 | | | |
| Fish 3 | 8.672.E-09 | | | | 9.989.E-01 | | | | 4.954.E-03 | | | |
| m. Figure 5p, q, Extended Data Figure 6a (Wall rotation) (one-sided Wilcoxon signed-rank test) | | | | | | | | | | | | |
|  | PF cor. | | | | PV cor. | | | | PF shift | | | |
| Direct comparsion | | | | | | | | | | | | |
| Fish 1 | 2.953.E-01 | | | | 1.708.E-01 | | | | 8.098.E-06 | | | |
| Fish 2 | 7.281.E-149 | | | | 5.993.E-130 | | | | 5.537.E-13 | | | |
| Fish 3 | 4.700.E-39 | | | | 2.577.E-16 | | | | 3.053.E-21 | | | |
| Fish 4 | 1.011.E-22 | | | | 4.790.E-54 | | | | 3.624.E-01 | | | |
| Rotation corrected | | | | | | | | | | | | |
| Fish 1 | 1.211.E-53 | | | | 2.510.E-34 | | | | 3.889.E-27 | | | |
| Fish 2 | 8.486.E-200 | | | | 9.162.E-131 | | | | 1.134.E-31 | | | |
| Fish 3 | 1.351.E-159 | | | | 1.770.E-118 | | | | 5.674.E-29 | | | |
| Fish 4 | 1.450.E-38 | | | | 3.275.E-90 | | | | 7.079.E-07 | | | |
| n. Figure 6d, Extended Data Figure 6a (one-sided Wilcoxon signed-rank test) | | | | | | | | | | | | |
|  | PF cor. | | | | PV cor. | | | | PF shift | | | |
| Fish 1 | 1.623.E-159 | | | | 2.479.E-135 | | | | 1.154.E-13 | | | |
| Fish 2 | 3.817.E-95 | | | | 9.149.E-137 | | | | 5.219.E-05 | | | |
| Fish 3 | 7.103.E-44 | | | | 8.437.E-137 | | | | 2.158.E-05 | | | |
| Fish 4 | 1.214.E-111 | | | | 1.800.E-141 | | | | 1.109.E-01 | | | |
| o. Figure 6e (one-sided Wilcoxon signed-rank test) | | | | | | | | | | | | |
| Fish 1 | 2.330 E-155 | | | |  | | | |  | | | |
| Fish 2 | 1.372 E-99 | | | |  | | | |  | | | |
| Fish 3 | 2.582 E-67 | | | |  | | | |  | | | |
| Fish 4 | 5.460 E-90 | | | |  | | | |  | | | |
| p. Extended Data Figure 8e (Chamber rotation) (one-sided Wilcoxon signed-rank test) | | | | | | | | | | | | |
|  | PF cor. | | | | PV cor. | | | | PF shift | | | |
| Fish 1 | 1.000.E+00 | | | | 1.000.E+00 | | | | 1.000.E+00 | | | |
| Fish 2 | 8.181.E-15 | | | | 3.273.E-38 | | | | 1.511.E-02 | | | |
| Fish 3 | 1.000.E+00 | | | | 1.000.E+00 | | | | 9.998.E-01 | | | |
| q. Extended Data Figure 8e (Landmark removal) (one-sided Wilcoxon signed-rank test) | | | | | | | | | | | | |
|  | PF cor. | | | | PV cor. | | | | PF shift | | | |
| Fish 1 | 5.259.E-07 | | | | 9.651.E-01 | | | | 6.908.E-05 | | | |
| Fish 2 | 1.000.E+00 | | | | 1.000.E+00 | | | | 9.800.E-01 | | | |
| Fish 3 | 4.174.E-01 | | | | 9.283.E-01 | | | | 3.256.E-01 | | | |
| r. Extended Data Figure 8e (Wall morphing) (one-sided Wilcoxon signed-rank test) | | | | | | | | | | | | |
|  | PF cor. | | | | PV cor. | | | | PF shift | | | |
| Fish 1 | 4.847.E-03 | | | | 6.322.E-41 | | | | 4.096.E-03 | | | |
| Fish 2 | 9.994.E-01 | | | | 1.000.E-04 | | | | 7.130.E-01 | | | |
| Fish 3 | 4.447.E-25 | | | | 2.337.E-01 | | | | 3.946.E-12 | | | |
| Fish 4 | 6.410.E-12 | | | | 1.156.E-03 | | | | 3.375.E-02 | | | |
| s. Extended Data Figure 8e (Wall rotation) (one-sided Wilcoxon signed-rank test) | | | | | | | | | | | | |
|  | PF cor. | | | | PV cor. | | | | PF shift | | | |
| Fish 1 | 9.966.E-01 | | | | 9.998.E-01 | | | | 2.005.E-02 | | | |
| Fish 2 | 1.275.E-110 | | | | 1.541.E-125 | | | | 7.521.E-11 | | | |
| Fish 3 | 1.262.E-38 | | | | 3.787.E-34 | | | | 1.310.E-23 | | | |
| Fish 4 | 3.471.E-21 | | | | 2.425.E-47 | | | | 7.352.E-01 | | | |
| t. Extended Data Figure 8e (Fish removal) (one-sided Wilcoxon signed-rank test) | | | | | | | | | | | | |
|  | PF cor. | | | | PV cor. | | | | PF shift | | | |
| Fish 1 | 1.000.E+00 | | | | 1.000.E+00 | | | | 8.954.E-01 | | | |
| Fish 2 | 7.177.E-54 | | | | 1.376.E-127 | | | | 8.614.E-06 | | | |
| Fish 3 | 3.940.E-12 | | | | 3.447.E-03 | | | | 1.000.E+00 | | | |
| Fish 4 | 6.742.E-38 | | | | 1.545.E-95 | | | | 2.236.E-02 | | | |
| u. Extended Data Figure 8e (Landmark removal+ Fish removal) (one-sided Wilcoxon signed-rank test) | | | | | | | | | | | | |
|  | PF cor. | | | | PV cor. | | | | PF shift | | | |
| Fish 1 | 3.858.E-08 | | | | 7.893.E-113 | | | | 4.794.E-02 | | | |
| Fish 2 | 6.644.E-31 | | | | 2.606.E-106 | | | | 2.781.E-01 | | | |
| Fish 3 | 1.125.E-40 | | | | 1.249.E-94 | | | | 4.178.E-04 | | | |
| Fish 4 | 1.961.E-22 | | | | 8.904.E-07 | | | | 4.627.E-07 | | | |
| v. Extended Data Figure 8e (Wall morphing + Fish removal) (one-sided Wilcoxon signed-rank test) | | | | | | | | | | | | |
|  | PF cor. | | | | PV cor. | | | | PF shift | | | |
| Fish 1 | 2.601.E-06 | | | | 2.116.E-38 | | | | 9.991.E-01 | | | |
| Fish 2 | 3.649.E-10 | | | | 8.064.E-43 | | | | 2.003.E-01 | | | |
| Fish 3 | 9.174.E-12 | | | | 8.583.E-01 | | | | 2.290.E-04 | | | |
| w. Extended Data Figure 8e (Wall morphing + Landmark removal + Fish removal) (one-sided Wilcoxon signed-rank test) | | | | | | | | | | | | |
|  | PF cor. | | | | PV cor. | | | | PF shift | | | |
| Fish 1 | 7.746.E-163 | | | | 2.479.E-135 | | | | 6.106.E-14 | | | |
| Fish 2 | 2.338.E-93 | | | | 7.496.E-135 | | | | 3.206.E-02 | | | |
| Fish 3 | 1.177.E-48 | | | | 8.437.E-137 | | | | 9.439.E-06 | | | |
| Fish 4 | 1.491.E-101 | | | | 2.582.E-141 | | | | 5.672.E-04 | | | |
| x. Extended Data Figure 9e (one-sided Mann-Whitney U test) | | | | | | | | | | | | |
|  | PF cor. | | | | PV cor. | | | | PF shift | | | |
|  | A1-A2 vs. A1-B | | A1-B vs. B-A2 | | A1-A2 vs. A1-B | | A1-B vs. B-A2 | | A1-A2 vs. A1-B | | A1-B vs. B-A2 | |
| Fish 1 | 1.142.E-79 | | 1.000.E+00 | | 4.341.E-31 | | 1.000.E+00 | | 1.395.E-12 | | 9.998.E-01 | |
| Fish 2 | 1.170.E-23 | | 9.148.E-01 | | 1.293.E-16 | | 6.340.E-01 | | 3.836.E-01 | | 7.574.E-01 | |
| Fish 3 | 6.632.E-58 | | 8.726.E-01 | | 6.011.E-117 | | 1.161.E-01 | | 1.678.E-07 | | 2.416.E-01 | |
| Fish 4 | 2.886.E-130 | | 1.171.E-01 | | 5.150.E-191 | | 2.574.E-34 | | 1.065.E-20 | | 1.470.E-01 | |
| y. Extended Data Figure 10, PF correlation improvement after Nonrigid transformation with the best rotation angle, (non-parametric shuffle test (**Methods**)) | | | | | | | | | | | | |
|  | Wall morphing | | | | Wall morphing + Fish removal | | | | Wall morphing + Landmark removal + Fish removal | | | |
| Fish 1 | 3.070.E-01 | | | | 2.740.E-01 | | | | 7.000.E-03 | | | |
| Fish 2 | 1.000.E+00 | | | | 0.000.E+00 | | | | 0.000.E+00 | | | |
| Fish 3 | 0.000.E+00 | | | | 1.000.E+00 | | | | 5.400.E-02 | | | |
| Fish 4 | 9.990.E-01 | | | | NA | | | | 0.000.E+00 | | | |
| z. Extended Data Figure. 9e sample size | | | | | | | | | | | | |
|  | PF cor., A1-A2 | PF cor., A1-B | | PF cor., B-A2 | PV cor., A1-A2 | PV cor., A1-B | | PV cor., B-A2 | PF shift, A1-A2 | PF shift, A1-B | | PF shift, B-A2 |
| Fish 1 | 1281 | 1072 | | 1096 | 793 | 744 | | 767 | 221 | 199 | | 217 |
| Fish 2 | 391 | 514 | | 294 | 797 | 757 | | 757 | 24 | 52 | | 58 |
| Fish 3 | 851 | 876 | | 232 | 791 | 535 | | 520 | 31 | 58 | | 8 |
| Fish 4 | 1100 | 1022 | | 992 | 812 | 652 | | 653 | 188 | 304 | | 182 |
