# Supplementary material for: A population code for spatial representation in the zebrafish telencephalon
Source: Nature. 2024 Aug 28;634(8033):397–406. doi: 10.1038/s41586-024-07867-2 (PMC11464381; doi:10.1038/s41586-024-07867-2)
Supplement: Supplementary file 2 — Reporting Summary [file 41586_2024_7867_MOESM2_ESM.pdf]

nature portfolio

Corresponding author(s): Drew N Robson & Jennifer M Li

Last updated by author(s): Jun 13, 2024

Reporting Summary

Nature Portfolio wishes to improve the reproducibility of the work that we publish. This form provides structure for consistency and transparency in reporting. For further information on Nature Portfolio policies, see our Editorial Policies and the Editorial Policy Checklist.

Statistics

For all statistical analyses, confirm that the following items are present in the figure legend, table legend, main text, or Methods section.

n/a	Confirmed	
☐	☒	The exact sample size (*n*) for each experimental group/condition, given as a discrete number and unit of measurement
☐	☒	A statement on whether measurements were taken from distinct samples or whether the same sample was measured repeatedly
☐	☒	The statistical test(s) used AND whether they are one- or two-sided<br>*Only common tests should be described solely by name; describe more complex techniques in the Methods section.*
☒	☐	A description of all covariates tested
☐	☒	A description of any assumptions or corrections, such as tests of normality and adjustment for multiple comparisons
☐	☒	A full description of the statistical parameters including central tendency (e.g. means) or other basic estimates (e.g. regression coefficient) AND variation (e.g. standard deviation) or associated estimates of uncertainty (e.g. confidence intervals)
☐	☒	For null hypothesis testing, the test statistic (e.g. *F*, *t*, *r*) with confidence intervals, effect sizes, degrees of freedom and *P* value noted<br>*Give P values as exact values whenever suitable.*
☒	☐	For Bayesian analysis, information on the choice of priors and Markov chain Monte Carlo settings
☒	☐	For hierarchical and complex designs, identification of the appropriate level for tests and full reporting of outcomes
☒	☐	Estimates of effect sizes (e.g. Cohen's *d*, Pearson's *r*), indicating how they were calculated

Our web collection on statistics for biologists contains articles on many of the points above.

Software and code

Policy information about availability of computer code

Data collection

Software for data collection was written in Rust and CUDA C. GPU processing was implemented for fish tracking, as well as online image registration for z tracking. Data collection code is already available from our previous publications.

Data analysis

All software for data analysis was written in Julia 1.10 and Python (Scikit-learn 1.5.0) using the package ecosystem for both languages, Data analysis code will be available online upon publication at https://github.com/RoLiLab/fish_place_cell_Yang_2024. All software for behavior and image analysis, as well as license information and implementation notes, will be available upon request upon publication.

For manuscripts utilizing custom algorithms or software that are central to the research but not yet described in published literature, software must be made available to editors and reviewers. We strongly encourage code deposition in a community repository (e.g. GitHub). See the Nature Portfolio guidelines for submitting code & software for further information.

Data

Policy information about availability of data

All manuscripts must include a data availability statement. This statement should provide the following information, where applicable:

- Accession codes, unique identifiers, or web links for publicly available datasets
- A description of any restrictions on data availability
- For clinical datasets or third party data, please ensure that the statement adheres to our policy

The data that support the findings of this study are available from the corresponding authors upon reasonable request.

Human research participants

Policy information about studies involving human research participants and Sex and Gender in Research.

Reporting on sex and gender	There were no human research participants.
Population characteristics	N/A
Recruitment	N/A
Ethics oversight	N/A

Note that full information on the approval of the study protocol must also be provided in the manuscript.

Field-specific reporting

Please select the one below that is the best fit for your research. If you are not sure, read the appropriate sections before making your selection.

☒ Life sciences ☐ Behavioural & social sciences ☐ Ecological, evolutionary & environmental sciences

For a reference copy of the document with all sections, see nature.com/documents/nr-reporting-summary-flat.pdf

Life sciences study design

All studies must disclose on these points even when the disclosure is negative.

Sample size	A sample size of 3-11 animals was used depending on the experimental condition or analysis method. A total of 42 animals was used.
Data exclusions	Fish were excluded from the analyses, if quiescent for more than 20 minutes of the experiment. In such case there is not sufficient data to reliably construct the place maps.
Replication	N/A
Randomization	N/A
Blinding	N/A

Reporting for specific materials, systems and methods

We require information from authors about some types of materials, experimental systems and methods used in many studies. Here, indicate whether each material, system or method listed is relevant to your study. If you are not sure if a list item applies to your research, read the appropriate section before selecting a response.

Materials & experimental systems

n/a	Involved in the study	
☒	☐	Antibodies
☒	☐	Eukaryotic cell lines
☒	☐	Palaeontology and archaeology
☐	☒	Animals and other organisms
☒	☐	Clinical data
☒	☐	Dual use research of concern

Methods

n/a	Involved in the study	
☒	☐	ChIP-seq
☒	☐	Flow cytometry
☒	☐	MRI-based neuroimaging

Animals and other research organisms

Policy information about studies involving animals; ARRIVE guidelines recommended for reporting animal research, and Sex and Gender in Research

Laboratory animals	All experiments in rectangular chambers and the ABA experiments used Tg(elavl3:H2B-GCaMP6s+/+) with nacre (mitfa-/-) at 6-9 dpf. The rest of the experiments used Tg(elavl3:H2B-GCaMP8s+/+ or GCaMP8s+/-) with nacre (mitfa-/-) at 6-13 dpf. In three of the rectangle to circle experiments, the fish expressed an additional allele of Tg(elavl3:GCaMP6s). Fish were reared on a 14/10 hr light/

	dark cycle at 25°C. They were maintained in sets of 20 in E3 water and were fed dry food daily.
Wild animals	No wild animal is used in this study.
Reporting on sex	zebrafish larvae (6-13 dpf). Sexual differentiation has not occurred by this age.
Field-collected samples	No field collected sample was used in this study.
Ethics oversight	Experiments are carried out in accordance with the Animal Welfare Office at the University of Tübingen and the Regierungspräsidium.

Note that full information on the approval of the study protocol must also be provided in the manuscript.
